# The Clinical Presentation of Culture-positive and Culture-negative, Quantitative Polymerase Chain Reaction (qPCR)-Attributable Shigellosis in the Global Enteric Multicenter Study and Derivation of a *Shigella* Severity Score: Implications for Pediatric *Shigella* Vaccine Trials

**DOI:** 10.1093/cid/ciaa1545

**Published:** 2020-10-12

**Authors:** Patricia B Pavlinac, James A Platts-Mills, Kirkby D Tickell, Jie Liu, Jane Juma, Furqan Kabir, Joseph Nkeze, Catherine Okoi, Darwin J Operario, Jashim Uddin, Shahnawaz Ahmed, Pedro L Alonso, Martin Antonio, Stephen M Becker, Robert F Breiman, Abu S G Faruque, Barry Fields, Jean Gratz, Rashidul Haque, Anowar Hossain, M Jahangir Hossain, Sheikh Jarju, Farah Qamar, Najeeha Talat Iqbal, Brenda Kwambana, Inacio Mandomando, Timothy L McMurry, Caroline Ochieng, John B Ochieng, Melvin Ochieng, Clayton Onyango, Sandra Panchalingam, Adil Kalam, Fatima Aziz, Shahida Qureshi, Thandavarayan Ramamurthy, James H Roberts, Debasish Saha, Samba O Sow, Suzanne E Stroup, Dipika Sur, Boubou Tamboura, Mami Taniuchi, Sharon M Tennant, Anna Roose, Deanna Toema, Yukun Wu, Anita Zaidi, James P Nataro, Myron M Levine, Eric R Houpt, Karen L Kotloff

**Affiliations:** 1 Department of Global Health, University of Washington, Seattle, Washington,USA; 2 Division of Infectious Diseases and International Health, University of Virginia, Charlottesville, Virginia,USA; 3 Center for Global Health Research, Kenya Medical Research Institute (KEMRI), Kenya; 4 Department of Paediatrics and Child Health, Aga Khan University, Karachi,Pakistan; 5 Center for Vaccine Development and Global Health, University of Maryland School of Medicine, Baltimore, Maryland,USA; 6 Medical Research Council Unit The Gambia at the London School of Hygiene and Tropical Medicine, Banjul,The Gambia; 7 International Centre for Diarrhoeal Disease Research, Bangladesh (ICDDR, B), Dhaka,Bangladesh; 8 Barcelona Centre for International Health Research (CRESIB), Hospital Clínic, Universitat de Barcelona, Barcelona,Spain; 9 Centro de Investigação em Saúde da Manhiça, Maputo,Mozambique; 10 Science Applications International Corporation (SAIC), Richmond, Virginia,USA; 11 Department of Global Health, Rollins School of Public Health, Emory University, Atlanta, Georgia, USA; 12 Global Disease Detection Division, Kenya Office of the US Centers for Disease Control and Prevention , Nairobi, Kenya; 13 Public Health Sciences, University of Virginia, Charlottesville, Virginia,USA; 14 National Institute of Cholera and Enteric Diseases, Kolkata,India; 15 Centre pour le Développement des Vaccins, Bamako,Mali; 16 Department of Medicine, University of Maryland School of Medicine, Baltimore, Maryland,USA; 17 Sanofi Pasteur, Swiftwater, Pennsylvania,USA; 18 Bill and Melinda Gates Foundation, Seattle, Washington,USA; 19 Department of Pediatrics, University of Maryland School of Medicine, Baltimore, Maryland,USA

## Abstract

**Background:**

*Shigella* is a leading cause of childhood diarrhea and target for vaccine development. Microbiologic and clinical case definitions are needed for pediatric field vaccine efficacy trials.

**Methods:**

We compared characteristics of moderate to severe diarrhea (MSD) cases in the Global Enteric Multicenter Study (GEMS) between children with culture positive *Shigella* to those with culture-negative, quantitative polymerase chain reaction (qPCR)-attributable *Shigella* (defined by an *ipaH* gene cycle threshold <27.9). Among *Shigella* MSD cases, we determined risk factors for death and derived a clinical severity score.

**Results:**

Compared to culture-positive *Shigella* MSD cases (n = 745), culture-negative/qPCR-attributable *Shigella* cases (n = 852) were more likely to be under 12 months, stunted, have a longer duration of diarrhea, and less likely to have high stool frequency or a fever. There was no difference in dehydration, hospitalization, or severe classification from a modified Vesikari score. Twenty-two (1.8%) *Shigella* MSD cases died within the 14-days after presentation to health facilities, and 59.1% of these deaths were in culture-negative cases. Age <12 months, diarrhea duration prior to presentation, vomiting, stunting, wasting, and hospitalization were associated with mortality. A model-derived score assigned points for dehydration, hospital admission, and longer diarrhea duration but was not significantly better at predicting 14-day mortality than a modified Vesikari score.

**Conclusions:**

A composite severity score consistent with severe disease or dysentery may be a pragmatic clinical endpoint for severe shigellosis in vaccine trials. Reliance on culture for microbiologic confirmation may miss a substantial number of *Shigella* cases but is currently required to measure serotype specific immunity.

## Teaser

Molecular methods identified *Shigella* more commonly than microbiologic culture in younger and stunted children. A simplified clinical score containing dehydration, hospitalization, and diarrhea duration could be used to stratify vaccine trial endpoints by severity based on its ability to predict death.

*Shigella* is a leading cause of diarrhea among children <5 years in resource-limited settings, associated with over 60 000 deaths in this age group per year [1]. Several *Shigella* vaccines are currently in development [2–4], and vaccine efficacy will be determined by the number of clinically relevant, *Shigella-*attributable diarrhea cases prevented by the vaccine [5].

Microbiological culture is the gold standard for *Shigella-*confirmation; however, the application of highly sensitive molecular tools, such as quantitative polymerase chain reaction (qPCR), has revealed a large burden of *Shigella* infections undetected by culture [6, 7]. This increased sensitivity of qPCR could make vaccine trials more efficient, but the clinical significance of culture-negative and PCR-attributable shigellosis is unclear.

Rotavirus vaccine is most efficacious in preventing severe rotavirus diarrhea [8], and the Vesikari score is commonly used to stratify clinical endpoints in vaccine trials [9]. There is no universally accepted clinical severity score for *Shigella* diarrhea in children, and the Vesikari score does not include severity indicators that may be specific to shigellosis, such as dysentery [5]. Identifying the ideal severity score to use in *Shigella* vaccine trials requires an empiric assessment of the performance of existing and new severity scores in disaggregating severe versus nonsevere cases of *Shigella* diarrhea.

The Global Enteric Multicenter Study (GEMS) was a multicountry case-control study that enrolled children seeking care for moderate-to-severe (MSD) diarrhea and matched controls [10]. Utilizing clinical and laboratory data from GEMS cases, we sought to inform a microbiologic and clinical case definition for severe, laboratory-confirmed *Shigella* diarrhea by answering 3 questions: (1) Does the clinical presentation of *Shigella* differ by culture versus qPCR? (2) What are risk factors for death among children with *Shigella*- attributed diarrhea? (3) Can we develop a clinical severity score specific to *Shigella,* and how does it compare to a modified Vesikari score (MVS) previously created to fit these data?

## METHODS

### Parent Study

Children aged 0–59 months presenting to health centers in Bangladesh, India, Kenya, Mali, Mozambique, Pakistan, and The Gambia with diarrhea were screened for eligibility into GEMS between 2007 and 2011 as described elsewhere [10]. Eligible children were those with an acute episode of MSD defined as 1 or more of the following: dehydration (sunken eyes, loss of skin turgor, intravenous rehydration recommended), dysentery, or hospital admission [11]. At enrollment, clinical history and sociodemographic information were ascertained using a standardized questionnaire, whole stool samples were collected, and a physical exam was performed. Length/height, weight, and mid-upper arm circumference (MUAC) were measured at enrollment and at a single follow-up visit that was performed 60 days later (acceptable range 50–90 days) at which time vital status was also ascertained.

Stool samples were originally processed using conventional enteric pathogen detection methods as described elsewhere [12, 13], and a portion of samples stored at −80°C. For *Shigella* diagnosis by bacterial culture, stool samples were transported in cold storage in buffered glycerol saline (BGS) transport media and were inoculated onto MacConkey and xylose lysine desoxycholate agar. Suspected *Shigella* colonies were confirmed using triple-sugar iron, motility indole ornithine (MIO lysine decarboxylase media), citrate and urea biochemical typing media. A random subset of stored stool samples and samples from all fatal cases (if not included in the random subset) were also tested by qPCR using a 32-enteropathogen TaqMan Array Card [7]. Cycle thresholds (C_t_) required to detect the pathogen gene target, which are inversely related to nucleic acid quantity, of ≥35 were deemed negative. The gene amplified for *Shigella* detection, *ipaH*, is shared by enteroinvasive *Escherichia coli* (EIEC), but all *ipaH* detections were assumed to be *Shigella* based on metagenomic sequencing in a subset of samples [14].

### Nested Study

In this secondary data analysis, we excluded controls as well as cases who did not have both culture and qPCR results available and then grouped MSD cases by *Shigella* culture results. Among culture-negative children, we further divided low- and high-quantity infections using the *ipaH* C_t_ cutoff (<27.9) associated with an odds ratio of 2.0 in the original qPCR GEMS analysis [7]. This grouping led to 4 mutually exclusive categories: (1) *Shigella* negative (no detection by culture or qPCR); (2) culture-negative/qPCR-unattributable (culture negative and 27.9 ≤ *ipaH* C_t_ <35); (3) culture-negative/qPCR-attributable (culture negative and *ipaH* C_t_ <27.9); and (4) culture-positive (culture-positive *Shigella* irrespective of qPCR value). The clinical and demographic characteristics of these 4 categories were compared using prevalence ratios determined from Poisson regression with culture-positive *Shigella* as the reference group and each dichotomous covariate of interest modeled separately in a model including site (indicator variable) and age (continuous variable). Characteristics of interest, ascertained at MSD presentation, included age, site, sex, dysentery (visibly bloody stool reported by caregiver, clinician, or laboratory technician), mucoid stool, caregiver-reported number of days of diarrhea prior to presentation, caregiver reported number of loose stools in previous 24 hours, axillary temperature, caregiver-reported vomiting, clinician-determined dehydration status (according to World Health Organization [WHO] IMCI guidelines [15]), stunting (length for age z-score [LAZ] <−2), wasting (mid-upper arm circumference [MUAC] < 12.5cm among children 6 months or older), and admission to hospital. We utilized clinical signs at presentation to recreate the MVS generated previously with these data [16]. This MVS totaled 16 points and was categorized as mild (1–5 points), moderate (6–8 points), and severe (9–16 points). To establish the likelihood of causes of diarrhea other than *Shigella* in each of the 4 diagnostic categories, we considered site-and age-adjusted attributable fractions ≥ 0.5, derived from qPCR C_t_-values [17]. These pathogens were grouped as: viral (astrovirus, norovirus, rotavirus, sapovirus, adenovirus), parasitic (*Cryptosporidium*, *Entamoeba histolytica*, *Cyclospora*, *Isospora*), and other bacterial (*Aeromonas Campylobacter*, *Helicobacter pylori*, *Salmonella*, *Vibrio cholerae*, enteroaggregative Escherichia coli (E.coli) [EAEC], heat-stable enterotoxin-producing E. coli [ST-ETEC], heat-labile enterotoxin-producing E. coli [LT-ETEC], typical enteropathogenic E.coli [tEPEC], Shiga toxin producing E. coli [STEC]). The prevalence of other causes was compared between the 4 *Shigella* categories in age- and site-adjusted Poisson regression models.

To establish risk factors for death among children with *Shigella-*attributed diarrhea, we excluded children who were *Shigella* negative (by culture and qPCR) or had culture-negative/qPCR-unattributable *Shigella* and children without a 60-day follow-up visit in which vital status (and date of death, if applicable) was ascertained. Cox proportional hazards regression was used to identify univariate and adjusted risk factors for death in the first 14-days after presentation. We evaluated deaths in the 14-days to capture deaths most likely related to the MSD. Adjusted models included site (indicator variable) and age (continuous variable).

We derived a new severity score (model-derived score) based on risk of dying in the 14-days after MSD presentation using forward stepwise Cox proportional hazards regression and Akaike information criteria (AIC) for model-selection. The following clinical variables were considered in building this model: dysentery, mucoid stool, duration of diarrhea including and prior to the day of enrollment, maximum number of loose stools in last 24 hours, axillary temperature, caregiver-reported vomiting, WHO dehydration status, and clinician decision to hospitalize. Continuous variables were categorized to match that of the previously published MVS [16]. The final Cox model coefficients were used to calculate the new score using methods described elsewhere [18]. The total number of possible points were constrained to 16 and categorized as mild (<6), moderate [6–8], and severe (9+) to be consistent with the MVS [16].

The model-derived score and the MVS were compared using the area under the curve (AUC) calculated from a logistic model containing deaths in the first 14 days as the outcome and the continuous score as the independent variable with bootstrapped standard errors and a chi-square statistic. Finally, both scores’ ability to predict odds of death beyond 14-days (among those 14-day survivors) were also evaluated using logistic-regression based AUCs with bootstrapped standard errors.

To evaluate the robustness of our findings, we repeated all analyses in 3 subsets of data: (1) Excluding children with another possible etiology based on site and age-adjusted attributable fraction ≥0.5 in the culture-negative/qPCR-attributable and culture-positive groups; (2) excluding data from Bangladesh because of the uniquely high culture-positivity and dysentery rate at this site [7], and (3) excluding the fatal cases that were enriched in the sample (not qPCR-tested randomly).

Analyses were conducted in Stata 14.0 (Stata Corp, College Station, TX, USA) with an alpha of 0.05. The funder had no role in this manuscript’s design, data collection, analysis, writing, or submission.

## RESULTS

**Figure 1.**
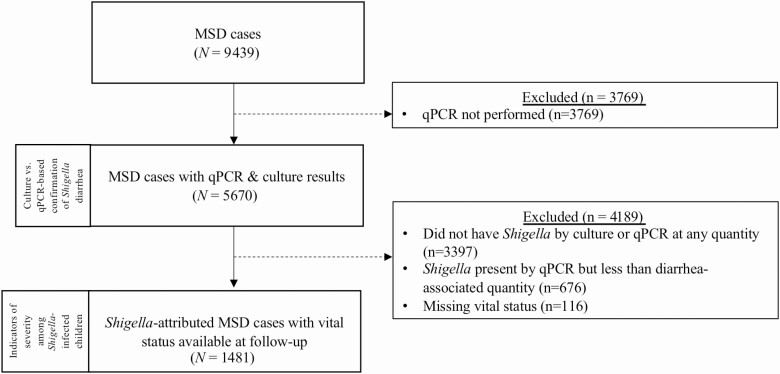
Participant flow of children with MSD included in each analysis. Abbreviations: MSD, moderate to severe diarrhea; qPCR, quantitative polymerase chain reaction.

Of 9439 MSD cases enrolled in GEMS, 5670 had qPCR results and were included in the analysis of clinical presentation by *Shigella* diagnostic assay (Figure 1). Sixty percent (n = 3397) of children did not have *Shigella* detected by culture or by qPCR at any C_t_ value. *Shigella* was isolated from culture in 745 children (13.1%), the majority (727 [97.5%]) of which were detected by qPCR (697 [95.9%] at qPCR-attributable levels and 30 [4.1%] qPCR-unattributable). Of the culture-positive *Shigella* cases, 65.4% were *S. flexneri*, 24.0% *S. sonnei*, 5.5% *S.dysenteriae*, and 5.1% *S*. *boydii*, as described elsewhere [12]. Of 4925 culture-negative MSD cases, qPCR-attributable *Shigella* infections were identified in an additional 852 (17.3%) children and qPCR-unattributable infections in 676 (13.7%).

### Culture Versus qPCR-Based Confirmation of *Shigella* Diarrhea

Accounting for potential confounding by age and site, compared to children with culture-positive shigellosis (Tables 1 and 2), cases with culture-negative/qPCR-attributable shigellosis were more likely to be under one year of age, stunted, and have had more than three days of diarrhea; they were also less likely to be febrile or have passed more than 6 loose stools in a day. The prevalence of a concomitant attributable viral pathogen was similar between culture-positive (13.3%) and culture-negative/qPCR-attributable (15.6%) shigellosis as was the likelihood of dysentery, mucoid stool, severe dehydration, vomiting, hospital admission, and a “severe” classification by the MVS. When removing episodes with other potentially attributable pathogens detected, the observed differences in clinical presentation of culture-positive and culture-negative/qPCR-attributable shigellosis did not change for any manifestation other than diarrhea duration, which became a more pronounced difference between the 2 diagnostic categories (Supplementary Table 1 [S1]). Exclusion of the Bangladesh site (Supplementary Table S2) resulted in a significantly lower prevalence of dysentery and negative/qPCR-attributable shigellosis compared to culture-positive shigellosis, and removal of fatal cases that were enriched in the data set (Supplementary Table S3) did not meaningfully change any comparisons.

**Table 1.** Frequencies of Sociodemographic, Clinical, and Pathogen Characteristics by Diagnostic Categories

		Absent						Present	
Characteristic	*Shigella* Culture	Absent^a^ (n = 3397)		qPCR-unattributable^b^ (n = 676)		qPCR-attributable^c^(n = 852)		Any qPCR value^d^ (n = 745)	
	*Shigella* qPCR	n	(%)	n	(%)	n	(%)	n	(%)
Sociodemographic									
Age									
1–11 m		1684	(49.6)	129	(19.1)	149	(17.5)	91	(12.2)
12–23 m		977	(28.8)	271	(40.1)	405	(47.5)	301	(40.4)
24–59 m		736	(21.7)	276	(40.8)	298	(35.0)	353	(47.4)
Female sex		1422	(42.5)	276	(40.8)	371	(43.5)	339	(45.5)
Site									
Bangladesh		357	(10.5)	35	(5.2)	98	(11.5)	410	(55.0)
India		567	(16.7)	123	(18.2)	138	(16.2)	58	(7.8)
Kenya		650	(19.3)	107	(15.8)	83	(9.7)	72	(9.7)
Mali		556	(16.4)	150	(22.2)	163	(19.1)	18	(2.4)
Mozambique		337	(9.9)	73	(10.8)	85	(10.0)	24	(3.2)
Pakistan		481	(14.2)	110	(16.3)	153	(18.0)	86	(11.5)
The Gambia		449	(13.2)	78	(11.5)	132	(15.5)	77	(10.3)
Clinical characteristics at enrollment									
Dysentery		459	(13.5)	91	(13.5)	355	(41.7)	534	(71.7)
Caregiver reported mucoid stool		765	(22.5)	148	(21.9)	255	(29.9)	364	(48.9)
Duration of diarrhea (including day of presentation) ≥3 d		1748	(51.5)	348	(51.5)	489	(57.4)	351	(47.1)
≥7 loose stools child in 24-h period		1213	(35.7)	218	(32.3)	303	(35.6)	424	(56.9)
Temperature ≥ 38°C		735	(21.6)	129	(19.1)	140	(16.4)	238	(32.0)
Caregiver reported vomiting > 3 times per day		1509	(44.4)	289	(42.8)	222	(26.1)	162	(21.7)
Severe dehydration		1908	(56.2)	390	(57.7)	437	(51.3)	221	(30.0)
Stunted (LAZ <−2)		943	(27.9)	240	(35.7)	310	(36.7)	214	(28.9)
Wasted^e^ (MUAC <12.5cm)		411	(14.3)	99	(15.3)	124	(15.0)	66	(9.1)
Hospitalized		819	(24.1)	158	(23.4)	145	(17.0)	209	(28.1)
Severe by modified Vesikari score^f^		1544	(45.5)	314	(46.5)	298	(35.0)	304	(40.8)
Other etiologies									
Viral^g^		1169	(34.4)	172	(25.4)	133	(15.6)	99	(13.3)
Parasitic^h^		280	(8.2)	41	(6.1)	54	(6.4)	15	(2.0)
Other bacteria^i^		615	(18.1)	168	(24.9)	185	(21.7)	61	(8.2)

Abbreviations: E. coli, Escherichia coli; EAEC, enteroaggregative Escherichia coli; LAZ, length for age z-score; LT-ETEC, heat-labile enterotoxin-producing E. coli; MUAC, mid-upper arm circumference; qPCR; quantitative polymerase chain reaction; STEC, Shiga toxin producing E. coli; ST-ETEC, heat-stable enterotoxin-producing E. coli; tEPEC, typical enteropathogenic E. coli.

^a^ *ipaH* C_t_ value ≥35.

^b^27.9 ≤ *ipaH* C_t_ <35.

^c^ *ipaH* C_t_ value <27.9.

^d^Absent by qPCR (n = 18 [2.4%]), present below diarrhea-associated quantity (n = 30 [4.0%]), present at, or above, diarrhea associated quantity (n = 697 [93.6%]).

^e^Among those ≥6 months of age (in whom MUAC is validated).

^f^As derived in Kotloff et al, Vaccine, 2017.

^g^Site and age-adjusted attributable fraction ≥.5 for any of the following: adenovirus, astrovirus, norovirus, rotavirus, sapovirus, adenovirus.

^h^Site and age-adjusted attributable fraction ≥.5 for any of the following: *Cryptosporidium*, *Entamoeba histolytica*, *Cyclospora*, *Isospora.*

^i^Site and age-adjusted attributable fraction ≥.5 for any of the following: *H. pylori*, *Campylobacter*, *Aeromonas, Salmonella*, *V. cholerae*, EAEC, ST-ETEC, LT-ETEC, tEPEC, STEC.

**Table 2.** Sociodemographic, Clinical, and Pathogen Factors Associated With *Shigella* Diagnostic Categories

		Absent						Present	
Characteristic	*Shigella* Culture	Absent^a^ (n = 3397)		qPCR-unattributable^b^(n = 676)		qPCR-attributable^c^(n = 852)		Any qPCR value^d^ (n = 745)	
	*Shigella* qPCR	aPR^e^	(95% CI)	aPR^e^	(95% CI)	aPR^e^	(95% CI)	aPR^e^	(95% CI)
Sociodemographic									
Age <12 m		4.52	(3.63–5.63)	1.76	(1.33–2.32)	1.57	(1.20–2.05)	Ref	…
Female sex		.89	(.78–1.01)	.85	(.72–1.01)	0.91	(.78–1.07)	Ref	…
Clinical characteristics at enrollment									
Dysentery		.37	(.32–.42)	.40	(.31–.50)	1.06	(.91–1.22)	Ref	…
Caregiver reported mucoid stool		.66	(.57–.77)	.72	(.58–.89)	0.95	(.80–1.13)	Ref	…
Duration of diarrhea ≥3 d		1.04	(.92–1.19)	1.09	(.93–1.28)	1.20	(1.04–1.39)	Ref	…
≥7 loose stools child in 24-h period		.83	(.73–.94)	.80	(.67–.95)	0.82	(.70–.96)	Ref	…
Temperature ≥ 38°C		.78	(.66–.92)	.71	(.56–.90)	0.62	(.50–.78)	Ref	…
Caregiver reported vomiting >3 times per day		1.84	(1.55–2.20)	1.85	(1.51–2.27)	1.13	(.91–1.39)	Ref	…
Severe dehydration		1.22	(1.05–1.41)	1.22	(1.03–1.45)	1.14	(.96–1.34)	Ref	…
Stunted (LAZ <−2)		1.05	(.89–1.24)	1.21	(.99–1.48)	1.27	(1.05–1.53)	Ref	…
Wasted^f^ (MUAC <12.5cm)		.86	(.65–1.14)	1.19	(.85–1.64)	1.08	(.79–1.48)	Ref	…
Hospitalized		1.29	(1.08–1.54)	1.37	(1.10–1.71)	0.90	(.72–1.13)	Ref	…-
Severe by modified Vesikari score^g^		1.11	(.95–1.25)	1.16	(.98–1.37)	0.87	(.74–1.03)	Ref	…
Other etiologies									
Viral^h^		2.24	(1.80–2.79)	2.56	(1.53–2.56)	1.18	(.91–1.55)	Ref	…
Parasitic^i^		1.94	(1.14–3.32)	2.93	(.88–2.93)	1.73	(.97–3.10)	Ref	…
Other bacteria^j^		2.24	(1.69–2.97)	3.88	(2.09–3.88)	2.54	(1.88–3.44)	Ref	…

Abbreviations: aPR, adjusted prevalence ratio; CI, confidence interval; E. coli, Escherichia coli; EAEC, enteroaggregative Escherichia coli; LAZ, length for age z-score; LT-ETEC, heat-labile enterotoxin-producing E. coli; MUAC, mid-upper arm circumference; qPCR; quantitative polymerase chain reaction; STEC, Shiga toxin producing E. coli; ST-ETEC, heat-stable enterotoxin-producing E. coli; tEPEC, typical enteropathogenic E. coli.

^a^ *ipaH* C_ t_ value ≥35

^b^27.9 ≤ *ipaH* C_t_ <35

^c^ *ipaH* C_t_ value <27.9

^d^Absent by qPCR (n = 18 [2.4%]), present below diarrhea-associated quantity (n = 30 {4.0%}), present at, or above, diarrhea associated quantity (n = 697 [93.6%]).

^e^ aPR from relative risk regression assuming Poisson distribution adjusting for site (considered as an indicator variable) and age (considered continuously) except age model adjusted only for site.

^f^Among those ≥6 months of age (in whom MUAC is validated).

^g^As derived in Kotloff et al, Vaccine, 2017.

^h^Site and age-adjusted attributable fraction ≥.5 for any of the following: astrovirus, norovirus, rotavirus, sapovirus, adenovirus.

^i^Site and age-adjusted attributable fraction ≥.5 for any of the following: *Cryptosporidium*, *Entamoeba histolytica*, *Cyclospora*, *Isospora.*

^j^Site and age-adjusted attributable fraction ≥.5 for any of the following: *H. pylori*, *Campylobacter*, *Aeromonas, Salmonella*, *V. cholerae*, EAEC, ST-ETEC, LT-ETEC, tEPEC, STEC.

In contrast, children with culture-negative/qPCR-unattributable shigellosis were more likely than children with culture-positive shigellosis to present with vomiting, severe dehydration, to be hospitalized, and to have a viral etiology (Tables 1 and 2). Similar associations were found when comparing *Shigella* negative (no detection by culture or qPCR) cases to culture-positive shigellosis. Subset analyses revealed similar findings, except the analysis excluding Bangladesh data, which found no differences in fever, severe dehydration, or hospitalization when comparing culture-positive *Shigella* cases to the other 2 groups (Supplementary Tables S1–S3).

Bacterial causes other than *Shigella* were more common in all 3 culture-negative groups (21.7% qPCR-attributable, 24.9% qPCR-unattributable, and 18.1% absent) compared to culture-positive *Shigella* (8.2%, Tables 1 and 2). Subset analyses led to similar findings (Supplementary Tables S1–S3). These associations did not appear to be driven by a single bacterial pathogen (Supplementary Table S4*A*/*B*).

### Risk Factors for Death Among Children With *Shigella*-attributed Diarrhea

The clinical and demographic features of the 1481 children with either culture-negative/qPCR-attributable or culture-positive *Shigella* with known vital status at follow-up are presented, by age, in Table 3. Children <12 months comprised 14.9% of the *Shigella-*attributed cases and were less likely to present with dysentery and fever and more likely to present with vomiting and dehydration. This trend held true when limiting to qPCR-attributable cases only (Supplementary Table S5) and culture-positive shigellosis (Supplementary Table S6).

**Table 3.** Age-stratified Characteristics of *Shigella* MSD Cases Defined as Culture Positive or Quantitative Polymerase Chain Reaction (qPCR)-Attributable (n = 1481)

Characteristic	0–5 m (n = 35)		6–11 m (n = 185)		12–23 m (n = 654)		24–59 m (n = 607)	
	n	(%)	n	(%)	n	(%)	n	(%)
Sociodemographic								
Female sex	14	(40.0)	74	(40.0)	307	(46.9)	263	(43.3)
Site								
Bangladesh	3	(8.6)	49	(26.5)	198	(30.3)	252	(41.5)
India	5	(14.3)	19	(10.3)	62	(9.5)	101	(16.6)
Kenya	9	(25.7)	26	(14.1)	58	(8.9)	56	(9.2)
Mali	1	(2.9)	16	(8.7)	87	(13.3)	55	(9.1)
Mozambique	0	(0)	11	(6.0)	49	(7.5)	35	(5.8)
Pakistan	15	(42.9)	32	(17.3)	92	(14.1)	60	(9.9)
The Gambia	2	(5.7)	32	(17.2)	108	(16.5)	48	(7.9)
Clinical characteristics								
Dysentery	13	(37.1)	88	(47.6)	345	(52.8)	390	(64.3)
Caregiver reported mucoid stool	13	(37.1)	70	(37.8)	253	(38.7)	247	(40.7)
Duration of diarrhea (including day of presentation)								
1–3	19	(54.3)	128	(69.2)	460	(70.3)	448	(73.8)
4–5	14	(40.0)	35	(18.9)	143	(21.9)	124	(20.4)
6+	2	(5.7)	22	(11.9)	51	(7.8)	35	(5.8)
Max no. of loose stools child passed in 24-h period								
≤6	20	(57.1)	95	(51.4)	361	(55.2)	321	(52.9)
7–10	11	(31.4)	66	(35.7)	196	(30.0)	182	(30.0)
>10	4	(11.4)	24	(13.0)	97	(14.8)	104	(17.1)
Axillary temperature at presentation								
<38°C	30	(85.7)	152	(82.2)	510	(78.0)	431	(71.0)
38–38.9°C	3	(8.6)	24	(13.0)	80	(12.2)	106	(17.5)
≥39°C	2	(5.7)	9	(4.9)	64	(9.8)	70	(11.5)
Caregiver reported vomiting ≥3 times per day	12	(34.3)	63	(34.1)	159	(24.3)	124	(20.4)
WHO-defined dehydration categories								
None	11	(31.4)	53	(28.7)	208	(31.8)	255	(42.0)
Some	5	(14.3)	46	(24.9)	165	(25.2)	141	(23.2)
Severe	19	(54.3)	86	(46.5)	281	(43.0)	211	(34.8)
Modified Vesikari score^a^								
Mild	6	(17.1)	24	(13.0)	78	(11.9)	93	(15.3)
Moderate	15	(42.9)	81	(43.8)	326	(49.9)	299	(49.3)
Severe	14	(40.0)	80	(43.2)	250	(38.2)	215	(35.4)
Stunted (LAZ <−2)	14	(40.0)	46	(25.1)	197	(30.3)	220	(36.4)
Wasted (MUAC <12.5cm)^b^	--	--	50	(27.0)	97	(14.8)	27	(4.5)

Abbreviations: LAZ, length for age z-score; MSD, moderate to severe diarrhea; MUAC, mid-upper arm circumference; WHO, World Health Organization.

^a^As derived in Kotloff et al, Vaccine, 2017.

^b^Among children ≥6 months.

Forty-two children (2.8%) with *Shigella*-attributable diarrhea died during follow-up, of whom 22 (52.4%) died in the first 2 weeks. There was no difference in risk of death between culture negative/qPCR-attributable shigellosis and culture-positive shigellosis (adjusted hazard ratio [aHR]: 1.1, 95% confidence interval [CI]: .5–2.6). Age <6 months, duration of diarrhea >3 days, vomiting >3 times per day, stunting, MUAC under 12.5 cm, and hospital admission were associated with risk of death in the first 14 days (Table 4). Severe dehydration was not statistically associated with death in adjusted models, but 20/22 (90.9%) of the deaths among *Shigella* cases that occurred in the first 2 weeks were in children with severe dehydration. Dysentery was not associated with death in adjusted models, nor was presence of a second attributable etiology. When excluding episodes with additional potentially attributable pathogens (Supplementary Table S7), we found all risk factors remained significantly associated with death, including vomiting. There were no differences in significant risk factors in the subset of children excluding Bangladesh (Supplementary Table S8). Finally, excluding enriched fatal cases (Supplementary Table S9), young age and diarrhea duration were no longer significantly associated with death, although the direction and magnitude of association were similar to primary analyses.

**Table 4.** Characteristics of *Shigella* Moderate to Severe Diarrhea (MSD) Cases Defined as Culture Positive or Quantitative Polymerase Chain Reaction (qPCR)-Attributable (N = 1481) Who Died Versus Those Who Survived in the 14 Days Post enrollment

Characteristic	Died (n = 22)		Survived (n = 1459)		Hazard Ratio^b^ (95% CI)	Hazard Ratio (95% CI)^c^
	n	(%)^a^	n	(%)^a^		
Sociodemographic						
Age						
0–5 m	3	(13.6)	32	(2.2)	10.7 (2.6–45.0)	6.3 (1.4–27.5)
6–11 m	4	(18.2)	181	(12.4)	2.6 (.7–9.8)	1.6 (.4–5.8)
12–23 m	10	(45.5)	644	(44.1)	1.9 (.6–5.4)	1.3 (.4–3.8)
24–59 m	5	(22.7)	602	(41.3)	Ref	Ref
Sex						
Female	7	(31.8%)	651	(44.6%)	0.6 (.2–1.4)	0.6 (.2–1.4)
Male	15	(68.2%)	808	(55.4%)	Ref	Ref
Clinical characteristics						
Dysentery						
Present	7	(31.8%)	829	(56.8%)	0.4 (.2–.9)	0.6 (.2–1.6)
Absent	15	(68.2%)	630	(43.2%)	Ref	Ref
Caregiver reported mucoid stool						
Present	8	(36.4%)	575	(39.4%)	0.9 (.4–2.1)	1.2 (.5–3.0)
Absent	14	(63.6%)	884	(60.6%)	Ref	Ref
Duration of diarrhea (including day of presentation)						
≥3	19	(86.4%)	757	(51.9%)	5.8 (1.7–19.6)	4.4 (1.3–15.3)
<3	3	(13.6%)	702	(48.1%)	Ref	Ref
Max no. of loose stools child passed in 24-h period						
≥7	7	(31.8%)	677	(46.4%)	0.5 (.2–1.3)	1.1 (.4–2.9)
<7	15	(68.2%)	782	(53.6%)	Ref	Ref
Temperature						
≥38°C	9	(40.9%)	349	(23.9%)	2.2 (.9–5.1)	2.1 (.9–5.1)
<38°C	13	(59.1%)	1110	(76.1%)	Ref	Ref
Caregiver reported vomiting						
>3 times per day	12	(54.6%)	346	(23.7%)	3.8 (1.6–8.8)	2.5 (1.1–5.9)
≤3 times per day (or none)	10	(45.5%)	1113	(76.3%)	Ref	Ref
WHO-defined dehydration categories						
Severe	20	(90.9%)	577	(39.7%)	17.9 (2.4–133.3)	7.9 (.8–79.7)
Some	1	(4.6%)	356	(24.4%)	1.5 (.09–23.6)	1.3 (.07–23.5)
None	1	(4.6%)	526	(36.1%)	Ref	Ref
Chronic malnutrition						
Stunted (LAZ <−2)	11	(57.9%)	466	(32.1%)	2.9 (1.2–7.2)	3.0 (1.2–7.7)
Nonstunted	8	(42.1%)	988	(68.0%)	Ref	Ref
Acute malnutrition ^d^						
MUAC <12.5cm	8	(42.1%)	166	(11.6%)	5.4 (2.2–13.4)	3.3 (1.2–8.6)
MUAC ≥12.5cm	11	(57.9%)	1261	(88.4%)		Ref
Admission status at enrollment visit						
Hospitalized	16	(72.7%)	319	(21.9%)	9.3 (3.6–23.8)	14.5 (5.1–41.2)
Seen as outpatient	6	(27.3%)	1140	(78.1%)	Ref	Ref
Modified Vesikari score^e^						
Severe	18	(81.8%)	541	(37.1%)	5.9 (2.0–17.4)	4.4 (1.5–12.9)
Moderate	4	(18.2%)	717	(49.1%)	Ref	Ref
Mild	0	(0)	201	(13.8%)	Not estimable	Not estimable
Laboratory						
*Shigella* culture results						
Culture positive	9	(40.9%)	698	(47.8%)	0.8 (.3–1.8)	1.1 (.4–2.6)
Culture negative	13	(59.1%)	761	(52.2%)	Ref	Ref
*Shigella* qPCR C_t_ values ^f^						
<20.8	11	(50.0%)	635	(44.9%)	1.5 (.5–4.7)	1.9 (.6–6.0)
20.8–24.34	7	(31.8%)	436	(30.8%)	1.4 (.4–4.7)	1.7 (.5–5.8)
24.35–27.89	4	(18.2%)	343	(24.3%)	Ref	Ref
Other potential etiology^g^						
Yes	8	(36.4%)	429	(29.4%)	1.4 (.6–3.3)	1.4 (.6–3.3)
No	14	(63.6%)	1030	(70.6%)	Ref	Ref

Abbreviations: CI, confidence interval; E. coli, Escherichia coli; EAEC, enteroaggregative Escherichia coli; LAZ, length for age z-score; LT-ETEC, heat-labile enterotoxin-producing E. coli; MUAC, mid-upper arm circumference; STEC, Shiga toxin producing E. coli; ST-ETEC, heat-stable enterotoxin-producing E. coli; tEPEC, typical enteropathogenic E. coli; WHO, World Health Organization.

^a^Column percentages.

^b^From Cox proportional hazards regression including only the variable of interest in the model.

^c^From Cox proportional hazards regression including the variable of interest, site as an indicator variable, and age as a continuous variable except age model adjusted only for site.

^d^Among those ≥6 months of age in whom MUAC is validated.

^e^As derived in Kotloff et al, Vaccine, 2017.

^f^Among those with qPCR attributable-*Shigella* (n = 1436).

^g^Based on site and age-adjusted attributable fraction ≥0.5 for any of the following pathogens: astrovirus, norovirus, rotavirus, sapovirus, adenovirus, *Cryptosporidium*, *E. histolytica*, *Cyclospora*, *Isospora, isospora*, *H. pylori*, *Aeromonas Campylobacter*, *Salmonella*, *V. cholerae*, EAEC, ST-ETEC, LT-ETEC, tEPEC, STEC.

**Figure 2.**
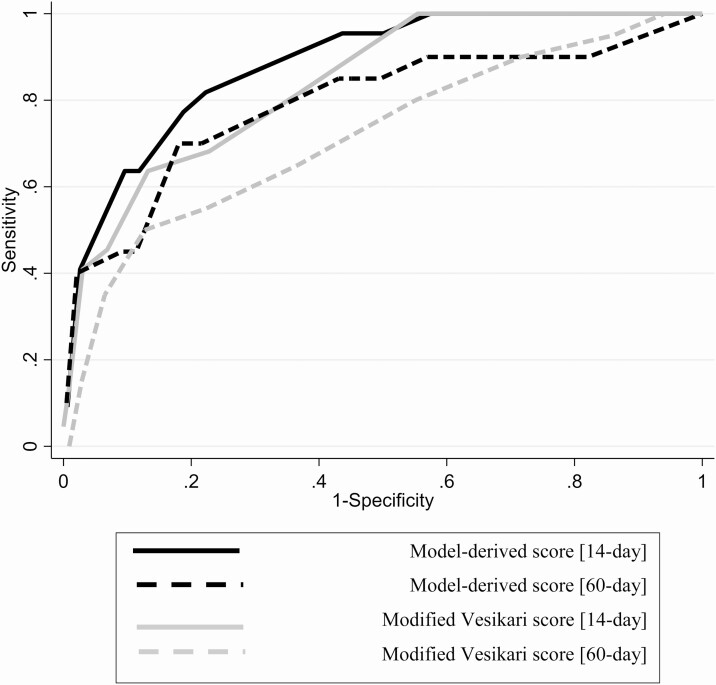
ROC curves of model-derived score and modified Vesikari score predicting death in first 14 days and within 60 days (range 50–90 days) among 14-day survivors. Model-derived score: AUC_0–14_ of 0.85 (95% CI: .77–.92); AUC_15–90_ 0.75 (95% CI: .60–.87). Modified Vesikari score: AUC_0–14:_ 0.80 (95% CI: .70–.88); AUC_15–90_: 0.67 (95% CI: .53–.80). Abbreviations: AUC, area under the curve; CI, confidence interval; ROC, receiver operating characteristic.

Based on model fit, 3 clinical features maximally predicted death: clinician decision to hospitalize, dehydration status, and diarrhea duration prior to presentation (Table 5, Supplementary Table S10). This model-derived score had an AUC of 0.85 for predicting 14-day mortality (95% CI: .76–.91). The MVS had a similar AUC (AUC = 0.80, 95% CI: .71–.88, *P*-value_AUC derived vs. AUC MVS_ = .077) (Figure 2). The model-derived score classified 564 (38.1%) of children as severe, 409 (27.6%) as moderate, and 729 (49.2%) as mild, whereas MVS classified 559 (37.7%) as severe, 721(48.7%) as moderate, and 201 (13.6%) as mild. Table 6 displays the median model-derived score and MVS across various characteristics. Against the outcome of death after 14 days among the 1459 14-day survivors, the 2 scores did not differ significantly (AUC_model_ = 0.75, 95% CI: .59–.87 vs AUC_MVS_: 0.67, 95% CI: .53–.80, *P* = .064). Among children ≥12 months (n = 1261) or in those with culture-confirmed *Shigella* (n = 707), the AUC values were not meaningfully different (Supplementary Figures S1 and S2) other than a significantly higher AUC for the model-derived score in predicting 14-day mortality (*P* = .048) in children aged 12 months or older.

**Table 5.** *Shigella* Model-derived Score and Modified Vesikari Score

Predictor	Model-derived Score	Modified Vesikari Score^a^
Days diarrhea prior to presentation (including day of presentation)		
≥6	3	3
4–5	2	2
1–3	0	1
Max no. of loose stools child passed in 24-h period		
>10	--	3
7–10	--	2
≤6	--	1
Max no. of vomiting episodes in 24-h period		
>3 times per day	--	2
≤3 times per day (or none)	--	1
WHO-defined dehydration categories		
Severe	8	3
Some	4	2
None	0	0
Temperature		
≥39°C	--	3
38.5–38.9°C	--	2
37.1–38.4°C	--	1
Hospitalized^b^		
Yes	5	2
No	0	0

Abbreviation: WHO, World Health Organization.

^a^As derived in Kotloff et al, Vaccine, 2017.

^b^In sum, 28 participants received intravenous (IV) rehydration in a short-stay ward, and these were upgraded to “hospitalized.”

**Table 6.** Median and Interquartile Range (IQR) of Model-derived and Modified Vesikari Scores by Participant Characteristic Among 1481 Children With *Shigella*-attributed Diarrhea

Characteristic	Model-derived Score		Modified Vesikari Score^a^	
	Median	(IQR)	Median	(IQR)
Sociodemographic				
Age				
0–5 m	8	(2–10)	8	(6–11)
6–11 m	8	(4–9)	8	(6–10)
12–23 m	6	(4–8)	8	(6–9)
24–59 m	5	(2–8)	8	(6–9)
Sex				
Female				
Male	6	(3–8)	8	(6–9)
Clinical characteristics	6	(3–8)	8	(6–9)
Dysentery				
Present	4	(0–8)	7	(6–9)
Absent	8	(5–10)	8	(7–10)
Caregiver reported mucoid stool				
Present	5	(2–8)	8	(6–9)
Absent	8	(4–9)	8	(7–9)
Duration of diarrhea (including day of presentation)				
≥3	6	(3–10)	8	(6–9)
<3	5	(4–8)	8	(6–10)
Max no. of loose stools child passed in 24-h period				
≥7	6	(4–8)	9	(7–10)
<7	5	(2–8)	7	(6–9)
Temperature				
≥38°C	6	(4–9)	10	(8–11)
<38°C	6	(2–8)	7	(6–9)
Caregiver reported vomiting				
>3 times per day	8	(5–10)	10	(8–11)
≤3 times per day (or none)	5	(2–8)	7	(6–9)
WHO-defined dehydration categories				
Severe	8	(8–13)	9	(8–11)
Some	4	(4–6)	7	(6–9)
None	2	(0–3)	6	(5–8)
Chronic malnutrition				
Stunted (LAZ <−2)	7	(3–9)	8	(7–9)
Nonstunted	5	(3–8)	8	(6–9)
Acute malnutrition ^b^				
MUAC <12.5 cm	8	(7–11)	9	(7–11)
MUAC ≥12.5 cm	5	(2–8)	8	(6–9)
Admission status at enrollment visit				
Hospitalized	9	(5–13)	10	(9–12)
Seen as outpatient	4	(2–8)	7	(6–8)
Modified Vesikari score^a^				
Severe	9	(8–13)	10	(9–11)
Moderate	4	(3–8)	7	(6–8)
Mild	0	(0–0)	5	(4, 5)
Laboratory				
*Shigella* culture results				
Culture positive	5	(2–8)	8	(6–10)
Culture negative	8	(4–9)	8	(6–9)
*Shigella* qPCR C_t_ values^c^				
<20.8	5	(3–8)	8	(6–10)
20.8–24.34	6	(2–8)	8	(6–9)
24.35–27.89	8	(4–9)	8	(6–9)
Other potential etiology^d^				
Yes	7	(4–9)	8	(7–9)
No	5	(2–8)	8	(6–9)

Abbreviations: CI, confidence interval; E. coli, Escherichia coli; EAEC, enteroaggregative Escherichia coli; IQR, interquartile range; LAZ, length for age z-score; LT-ETEC, heat-labile enterotoxin-producing E. coli; MUAC, mid-upper arm circumference; qPCR, quantitative polymerase chain reaction; STEC, Shiga toxin producing E. coli; ST-ETEC, heat-stable enterotoxin-producing E. coli; tEPEC, typical enteropathogenic E. coli; V. cholerae, Vibrio cholerae; WHO, World Health Organization.

^a^As derived in Kotloff et al, Vaccine, 2017.

^b^Among those≥6 months of age in whom MUAC is validated.

^c^Among those with qPCR attributable *Shigella* (n = 1436).

^d^Based on site and age-adjusted attributable fraction ≥.5 for any of the following pathogens: astrovirus, norovirus, rotavirus, sapovirus, adenovirus, *Cryptosporidium*, *E. histolytica*, *Cyclospora*, *Isospora*, *H. pylori*, *Campylobacter*, *Salmonella*, *V. cholerae*, EAEC, ST-ETEC, LT-ETEC, tEPEC, STEC.

## Discussion

Our secondary analysis of children <5 years of age presenting to health centers with MSD found the clinical presentations of culture-positive shigellosis and culture-negative/qPCR-attributable shigellosis to differ in terms of fever, duration, and stool frequency but not in terms dehydration, vomiting, and MVS severity. Presuming culture-negative/qPCR-attributable *Shigella* infections are indeed *Shigella* diarrhea, as we routinely assume with culture-positive *Shigella*, culture missed half of *Shigella*-attributed MSD cases in this study, with a higher likelihood of missing the diagnosis in children <12 months and those who were stunted. Infants and malnourished children may require a lower inoculum to cause MSD which may go undetected by culture methods. Culture also missed over half of the *Shigella*-associated deaths. A simplified severity score, composed of dehydration status, diarrhea duration prior to presentation, and clinician decision to hospitalize performed similarly to a MVS at predicting mortality in the 2 weeks following *Shigella-*diarrhea presentation.

Although reliance on culture determination alone in vaccine trials will require larger trials, omission of culture-confirmation will not be conducive to *Shigella* serotyping, a critical consideration in assessing serotype-specific immunity. Antibiotic resistance determination in *Shigella* and other enteric bacteria, an important secondary endpoint of *Shigella* vaccine trials, will also require cultured isolates for interpretation. In the absence of methods for culture-independent serotyping and resistance testing, we suggest that vaccine trials be powered for culture-confirmation with a prespecified secondary molecular microbiologic endpoint that disaggregates low and high concentrations of *Shigella* DNA.

Care-seeking for diarrhea has been shown to correlate with severity, linear growth faltering, and mortality [19–21]. Medically attended diarrhea has been suggested as a key feature of vaccine trial endpoints [5] and, practically, centralizes clinical assessments of dehydration and other indicators of severity. With a 14-day *Shigella* case fatality rate of approximately 1.5%, an MSD definition among children seeking care (as used in GEMS) may be sufficient for clinical endpoint severity classification. Alternatively, the MVS or the simplified model-derived severity score could be used to stratify severity among care-seeking diarrhea cases. The 3 factors included in our model-derived score are all included in the MVS, and the 2 scores performed similarly at predicting immediate deaths, despite our score being derived within the same dataset in which its performance was validated. Dysentery did not end up in the model-derived score, possibly because of antibiotic management of dysentery according to WHO guidelines [22]. Despite this lack of association, we advocate for dysentery to be included in a severe *Shigella* case definition because it is a sign of intestinal inflammation and epithelial destruction, consequences of *Shigella* that likely lead to its long-term impact on growth.

Risk factors analyses revealed the host factors of young age, stunting, and wasting to be independently associated with death, as has been found previously [23–26]. The clinical presentation of *Shigella* in infants more commonly included vomiting, dehydration, and absence of dysentery, which is consistent with previous findings among infants in Bangladesh using culture-based diagnosis [23]. Decisions about when to introduce a *Shigella* vaccine must weigh the lower burden but higher risk of *Shigella* infection among infants against the difficulty of including new vaccinations in the early infant immunization schedule. Moreover, vaccination prior to 6 months may be more effective by predating nutritional deterioration attributed to *Shigella.*

There were a number of limitations to this analysis. Because of its exploratory nature, the limited number of deaths, as well as the use of AIC, rather than *P*-values, for developing a severity score, we did not account for multiple comparisons. We were limited by which and how clinical data were collected to inform the severity scores. For example, 2 previously validated MVS [27, 28] could not be generated with this data due to the unavailability or lack of finer categorization of some symptoms. Validation of our model-derived *Shigella* severity score in other cohorts where death or other poor outcomes are ascertained would strengthen this score’s utility and generalizability. We chose 14-day mortality as the gold standard measure of severity; however, children may have severe shigellosis and survive. We found bacteria other than *Shigella* to be more common in children with culture-negative/qPCR-attributable *Shigella* (but not viral etiologies) and cannot exclude the possibility that a small subset of this group of children had a bacterial etiology other than *Shigella.* Ultimately, vaccine trials that include both culture- and qPCR-based diagnostics can confirm culture-negative qPCR-attributable episodes are indeed *Shigella*.

The GEMS study design and extensive diagnostic testing provided a unique opportunity to compare clinical features of *Shigella* by diagnostic categories and to examine risk factors for death. A composite severity score consistent with severe disease or dysentery may be a pragmatic clinical endpoint (confirmed by culture and secondarily, by qPCR) in vaccine trials.

## Supplementary Data

Supplementary materials are available at *Clinical Infectious Diseases* online. Consisting of data provided by the authors to benefit the reader, the posted materials are not copyedited and are the sole responsibility of the authors, so questions or comments should be addressed to the corresponding author.

ciaa1545_suppl_Supplementary_Table_1

## Notes

***Acknowledgments.*** The authors thank the children who participated in these studies and their families, and the dedicated physicians, nurses, scientists, and staff at each study site for their dedication and outstanding performance of clinical and laboratory study activities.

***Financial support.*** This work was supported by the Bill & Melinda Gates Foundation (OPP1019093 and OPP1197861).

***Potential conflicts of interest.*** P. P. and K. K. received funding from the Bill & Melinda Gates Foundation for part of the submitted work and outside the submitted work; D. S. reports employment and shares with GSK Vaccines, outside the submitted work. S. T. reports grants from the Bill & Melinda Gates Foundation during the conduct of the study. All other authors have no potential conflicts. All authors have submitted the ICMJE Form for Disclosure of Potential Conflicts of Interest. Conflicts that the editors consider relevant to the content of the manuscript have been disclosed.
