# Supplementary material for: The Clinical Presentation of Culture-positive and Culture-negative, Quantitative Polymerase Chain Reaction (qPCR)-Attributable Shigellosis in the Global Enteric Multicenter Study and Derivation of a Shigella Severity Score: Implications for Pediatric Shigella Vaccine Trials
Source: Clin Infect Dis. 2020 Oct 12;73(3):e569–79. doi: 10.1093/cid/ciaa1545 (PMC8326551; doi:10.1093/cid/ciaa1545)
Supplement: ciaa1545_suppl_Supplementary_Table_1 [file ciaa1545_suppl_supplementary_table_1.docx]

| Table S1. Sociodemographic, clinical, and pathogen factors associated with *Shigella* diagnostic categories removing children with a possible etiology (AF>.5) other than *Shigella* in the two likely *Shigella* categories (*Shigella* culture positive or *Shigella* culture positive/ molecular attributable). Bolding indicates statistical significance at the alpha=0.05 level. | | | | | | | | | | |
| --- | --- | --- | --- | --- | --- | --- | --- | --- | --- | --- |
|  | | ***Shigella***  **Culture** | **Absent** | | | | | | **Present** | |
| **Characteristic** | | ***Shigella***  **qPCR** | **Absent^i^**  **(n=3,397)** | | **qPCR-unattributable^ii^**  **(n=676)** | | **Present at, or above diarrhea-associated quantity^iii^**  **(n=539)** | | **Any quantity or absent^iv^**  **(n=584)** | |
|  |  |  | aPR* | (95%CI) | aPR* | (95%CI) | aPR* | (95%CI) | aPR* | (95%CI) |
| **Sociodemographic** | | |  |  |  |  |  |  |  |  |
|  | Age < 12 m | | 5.24 | (4.04-6.79) | 2.04 | (1.50-2.78) | 1.60 | (1.15-2.24) | Ref | -- |
|  | Female sex | | 0.88 | (0.76-1.02) | 0.84 | (0.7-1) | 0.89 | (0.74-1.07) | Ref | -- |
| **Clinical characteristics at enrollment** | | |  |  |  |  |  |  |  |  |
|  | Observed or caregiver reported blood in stool | | 0.36 | (0.30-0.42) | 0.40 | (0.31-0.50) | 1.12 | (0.94-1.32) | Ref | -- |
|  | Caregiver reported mucoid stool | | 0.64 | (0.55-0.75) | 0.70 | (0.57-0.87) | 0.96 | (0.78-1.17) | Ref | -- |
|  | Duration of diarrhea ≥3 days | | 1.05 | (0.91-1.21) | 1.10 | (0.93-1.3) | 1.17 | (0.99-1.39) | Ref | -- |
|  | ≥7 loose stools child in 24 hour period | | 0.83 | (0.72-0.96) | 0.80 | (0.67-0.97) | 0.78 | (0.64-0.94) | Ref | -- |
|  | Temperature ≥38°C | | 0.75 | (0.63-0.91) | 0.69 | (0.54-0.88) | 0.68 | (0.53-0.87) | Ref | -- |
|  | Caregiver reported vomiting > 3 times per day | | 2.11 | (1.72-2.58) | 2.11 | (1.68-2.65) | 1.15 | (0.89-1.49) | Ref | -- |
|  | Severe dehydration | | 1.22 | (1.03-1.43) | 1.21 | (1.01-1.46) | 1.09 | (0.90-1.33) | Ref | -- |
|  | Stunted (LAZ<-2) | | 1.07 | (0.89-1.29) | 1.23 | (0.99-1.52) | 1.33 | (1.07-1.65) | Ref | -- |
|  | Wasted^v^ (MUAC < 12.5cm) | | 0.87 | (0.64-1.18) | 1.18 | (0.83-1.68) | 0.91 | (0.63-1.33) | Ref | -- |
|  | Hospitalized | | 1.37 | (1.13-1.66) | 1.44 | (1.13-1.82) | 0.81 | (0.61-1.06) | Ref | -- |

1. ipah C_t_ value ≥ 35
2. 27.9 ≤ *ipaH* C_t_ <35
3. ipah C_t_ value < 27.9
4. Absent by TAC (n=10 [1.7%]), present below diarrhea-associated quantity (n=24 [4.1%]), present at, or above, diarrhea associated quantity (n=550 [94.2%])
5. Among those >6 months of age (in whom MUAC is validated)

* Adjusted prevalence ratios (aPR) from relative risk regression assuming Poisson distribution adjusting for site (considered as an indicator variable) and age (considered continuously) except age model adjusted only for site

| Table S2. Sociodemographic, clinical, and pathogen factors associated with *Shigella* diagnostic categories removing Bangladesh site because of unique *Shigella* epidemiology. | | | | | | | | | | |
| --- | --- | --- | --- | --- | --- | --- | --- | --- | --- | --- |
|  | | ***Shigella***  **Culture** | **Absent** | | | | | | **Present** | |
| **Characteristic** | | ***Shigella***  **qPCR** | **Absent^i^**  **(n=3,040)** | | **qPCR-unattributable^ii^**  **(n=641)** | | **Present at, or above diarrhea-associated quantity^iii^**  **(n=754)** | | **Any quantity or absent^iv^**  **(n=335)** | |
|  |  |  | aPR* | (95%CI) | aPR* | (95%CI) | aPR* | (95%CI) | aPR* | (95%CI) |
| **Sociodemographic** | | |  |  |  |  |  |  |  |  |
|  | Age < 12 m | | 2.92 | (2.23-3.82) | 1.11 | (0.81-1.54) | 1.05 | (0.77-1.44) | Ref | -- |
|  | Female sex | | 0.87 | (0.74-1.03) | 0.83 | (0.68-1.01) | 0.88 | (0.73-1.06) | Ref | -- |
| **Clinical characteristics at enrollment** | | |  |  |  |  |  |  |  |  |
|  | Observed or caregiver reported blood in stool | | 0.20 | (0.16-0.24) | 0.24 | (0.18-0.32) | 0.74 | (0.61-0.90) | Ref | -- |
|  | Caregiver reported mucoid stool | | 0.57 | (0.46-0.71) | 0.65 | (0.50-0.85) | 0.78 | (0.61-0.99) | Ref | -- |
|  | Duration of diarrhea ≥3 days | | 1.04 | (0.88-1.23) | 1.08 | (0.90-1.31) | 1.16 | (0.96-1.39) | Ref | -- |
|  | ≥7 loose stools child in 24 hour period | | 0.80 | (0.67-0.95) | 0.76 | (0.61-0.94) | 0.81 | (0.66-0.99) | Ref | -- |
|  | Temperature ≥38°C | | 0.84 | (0.67-1.06) | 0.76 | (0.57-1.01) | 0.68 | (0.51-0.9) | Ref | -- |
|  | Caregiver reported vomiting > 3 times per day | | 1.76 | (1.41-2.2) | 1.76 | (1.38-2.26) | 1.13 | (0.88-1.46) | Ref | -- |
|  | Severe dehydration | | 1.13 | (0.97-1.32) | 1.13 | (0.95-1.35) | 1.09 | (0.91-1.29) | Ref | -- |
|  | Stunted (LAZ<-2) | | 1.16 | (0.94-1.44) | 1.36 | (1.08-1.73) | 1.41 | (1.12-1.78) | Ref | -- |
|  | Wasted^v^ (MUAC < 12.5cm) | | 0.81 | (0.59-1.09) | 1.11 | (0.78-1.57) | 1.02 | (0.73-1.42) | Ref | -- |
|  | Hospitalized | | 1.13 | (0.86-1.49) | 1.27 | (0.93-1.72) | 0.95 | (0.69-1.29) | Ref | -- |
| **Other etiologies** | | |  |  |  |  |  |  |  |  |
|  | Viral^vi^ | | 2.42 | (1.75-3.34) | 2.15 | (1.52-3.06) | 1.34 | (0.93-1.93) | Ref | -- |
|  | Parasitic^vii^ | | 2.02 | (1.13-3.63) | 1.69 | (0.88-3.22) | 1.78 | (0.95-3.34) | Ref | -- |
|  | Other bacteria^viii^ | | 2.09 | (1.47-2.98) | 2.62 | (1.79-3.81) | 2.43 | (1.67-3.53) | Ref | -- |

1. ipah C_t_ value ≥ 35
2. 27.9 ≤ *ipaH* C_t_ <35
3. ipah C_t_ value < 27.9
4. Absent by TAC (n=10 [1.7%]), present below diarrhea-associated quantity (n=14 [4.2%]), present at, or above, diarrhea associated quantity (n=311 [92.8%])
5. Among those >6 months of age (in whom MUAC is validated)
6. Site and age-adjusted attributable fraction ≥.5 for any of the following: astrovirus, norovirus, rotavirus, sapovirus, adenovirus
7. Site and age-adjusted attributable fraction ≥.5 for any of the following: *Cryptosporidium*, *Entamoeba histolytica*, *Cyclospora*, *Isospora*
8. Site and age-adjusted attributable fraction ≥.5 for any of the following: *H. pylori*, *Campylobacter*, *Aeromonas,* *Salmonella*, *V. cholerae*, EAEC, St-ETEC, Lt-ETEC, tEPEC, STEC

* Adjusted prevalence ratios (aPR) from relative risk regression assuming Poisson distribution adjusting for site (considered as an indicator variable) and age (considered continuously) except age model adjusted only for site

| Table S3. Sociodemographic, clinical, and pathogen factors associated with *Shigella* diagnostic categories removing those who were not part of the random subset selected for molecular analysis (aka enriched fatal cases) | | | | | | | | | | |
| --- | --- | --- | --- | --- | --- | --- | --- | --- | --- | --- |
|  | | ***Shigella***  **Culture** | **Absent** | | | | | | **Present** | |
| **Characteristic** | | ***Shigella***  **qPCR** | **Absent^i^**  **(n=3,368)** | | **qPCR-unattributable^ii^**  **(n=667)** | | **qPCR-attributable^iii^**  **(n=843)** | | **Any quantity or absent^iv^**  **(n=740)** | |
|  |  |  | aPR* | (95%CI) | aPR* | (95%CI) | aPR* | (95%CI) | aPR* | (95%CI) |
| **Sociodemographic** | | |  |  |  |  |  |  |  |  |
|  | Age < 24 m | | 4.59 | (3.68-5.73) | 1.73 | (1.31-2.30) | 1.55 | (1.18-2.03) | Ref | -- |
|  | Female sex | | 0.88 | (0.77-1.01) | 0.85 | (0.71-1) | 0.91 | (0.78-1.07) | Ref | -- |
| **Clinical characteristics at enrollment** | | |  |  |  |  |  |  |  |  |
|  | Observed or caregiver reported blood in stool | | 0.36 | (0.33-0.42) | 0.40 | (0.32-0.51) | 1.06 | (0.92-1.23) | Ref | -- |
|  | Caregiver reported mucoid stool | | 0.67 | (0.57-0.77) | 0.73 | (0.59-0.89) | 0.95 | (0.8-1.13) | Ref | -- |
|  | Duration of diarrhea ≥3 days | | 1.05 | (0.93-1.2) | 1.10 | (0.94-1.29) | 1.21 | (1.04-1.4) | Ref | -- |
|  | ≥7 loose stools child in 24 hour period | | 0.82 | (0.73-0.94) | 0.79 | (0.66-0.94) | 0.83 | (0.71-0.97) | Ref | -- |
|  | Temperature ≥38°C | | 0.78 | (0.66-0.93) | 0.71 | (0.56-0.9) | 0.63 | (0.5-0.78) | Ref | -- |
|  | Caregiver reported vomiting > 3 times per day | | 1.87 | (1.57-2.24) | 1.86 | (1.52-2.29) | 1.12 | (0.91-1.39) | Ref | -- |
|  | Severe dehydration | | 1.23 | (1.06-1.43) | 1.23 | (1.03-1.46) | 1.14 | (0.97-1.35) | Ref | -- |
|  | Stunted (LAZ<-2) | | 1.06 | (0.9-1.25) | 1.20 | (0.98-1.46) | 1.26 | (1.04-1.52) | Ref | -- |
|  | Wasted^v^ (MUAC < 12.5cm) | | 0.87 | (0.66-1.16) | 1.19 | (0.85-1.66) | 1.09 | (0.79-1.49) | Ref | -- |
|  | Hospitalized | | 1.30 | (1.09-1.55) | 1.39 | (1.11-1.74) | 0.89 | (0.71-1.12) | Ref | -- |
| **Other etiologies** | | |  |  |  |  |  |  |  |  |
|  | Viral^vi^ | | 2.24 | (1.8-2.8) | 2.00 | (1.54-2.59) | 1.20 | (0.92-1.57) | Ref | -- |
|  | Parasitic^vii^ | | 2.05 | (1.18-3.57) | 1.73 | (0.93-3.2) | 1.82 | (1-3.32) | Ref | -- |
|  | Other bacteria^viii^ | | 2.27 | (1.71-3.01) | 2.89 | (2.12-3.95) | 2.60 | (1.92-3.52) | Ref | -- |

1. ipah C_t_ value ≥ 35
2. 27.9 ≤ *ipaH* C_t_ <35
3. ipah C_t_ value < 27.9
4. Absent by TAC (n=18 [2.4%]), present below diarrhea-associated quantity (n=30 [4.1%]), present at, or above, diarrhea associated quantity (n=692 [93.5%])
5. Among those >6 months of age (in whom MUAC is validated)
6. Site and age-adjusted attributable fraction ≥.5 for any of the following: astrovirus, norovirus, rotavirus, sapovirus, adenovirus
7. Site and age-adjusted attributable fraction ≥.5 for any of the following: *Cryptosporidium*, *Entamoeba histolytica*, *Cyclospora*, *Isospora*
8. Site and age-adjusted attributable fraction ≥.5 for any of the following: *H. pylori*, *Campylobacter*, *Aeromonas,* *Salmonella*, *V. cholerae*, EAEC, St-ETEC, Lt-ETEC, tEPEC, STEC

* Adjusted prevalence ratios (aPR) from relative risk regression assuming Poisson distribution adjusting for site (considered as an indicator variable) and age (considered continuously) except age model adjusted only for site.

| Table S4a. Other potential etiologies by diagnostic category | | | | | | | | | | |
| --- | --- | --- | --- | --- | --- | --- | --- | --- | --- | --- |
|  | | **Shigella**  **Culture** | **Absent** | | | | | | **Present** | |
| **Characteristic** | | **Shigella**  **qPCR** | **Absent^i^**  **(n=3,397)** | | **qPCR-unattributable^ii^**  **(n=676)** | | **qPCR-attributable^iii^**  **(n=852)** | | **Any qPCR value^iv^**  **(n=745)** | |
|  |  |  | n | (%) | n | (%) | n | (%) | n | (%) |
| Viral | | |  |  |  |  |  |  |  |  |
|  | Adenovirus | | 171 | (5.1) | 28 | (3.9) | 23 | (2.8) | 18 | (2.4) |
|  | Astrovirus | | 77 | (2.3) | 17 | (2.4) | 14 | (1.7) | 7 | (1.0) |
|  | Norovirus | | 57 | (1.7) | 5 | (0.69) | 8 | (0.95) | 8 | (1.1) |
|  | Rotavirus | | 809 | (24.3) | 116 | (16.1) | 53 | (6.3) | 49 | (6.6) |
|  | Sapovirus | | 45 | (1.4) | 16 | (2.2) | 12 | (1.4) | 9 | (1.2) |
| Parasitic | | |  |  |  |  |  |  |  |  |
|  | *Cryptosporidium* | | 241 | (7.2) | 31 | (4.3) | 46 | (5.5) | 10 | (1.4) |
|  | *Cyclospora* | | 11 | (0.3) | 1 | (0.1) | 3 | (0.4) | 3 | (0.4) |
|  | *Entamoeba histolytica* | | 21 | (0.6) | 8 | (1.1) | 3 | (0.4) | 1 | (0.1) |
|  | *Isospora* | | 2 | (0.1) | 0 | (0) | 0 | (0) | 1 | (0.1) |
| Other bacteria | | |  |  |  |  |  |  |  |  |
|  | *Aeromonas* | | 23 | (0.7) | 7 | (1.0) | 25 | (3.0) | 8 | (1.1) |
|  | *Campylobacter* | | 69 | (2.1) | 8 | (1.1) | 5 | (0.6) | 6 | (0.8) |
|  | EAEC | | 0 | (0) | 1 | (0.1) | 0 | (0) | 0 | (0) |
|  | *H. pylori* | | 67 | (2.0) | 25 | (3.5) | 35 | (4.2) | 15 | (2.0) |
|  | Lt-ETEC | | 0 | (0) | 0 | (0) | 0 | (0) | 0 | (0) |
|  | *Salmonella* | | 50 | (1.5) | 11 | (1.5) | 13 | (1.6) | 1 | (0.1) |
|  | STEC | | 0 | (0) | 0 | (0) | 0 | (0) | 0 | (0) |
|  | St-ETEC | | 273 | (8.2) | 83 | (11.5) | 72 | (8.5) | 18 | (2.4) |
|  | typical EPEC | | 27 | (0.8) | 4 | (0.6) | 12 | (1.4) | 2 | (0.3) |
|  | *V. cholerae* | | 100 | (3.0) | 40 | (5.6) | 23 | (2.7) | 4 | (0.5) |

1. ipah C_t_ value ≥ 35
2. 27.9 ≤ *ipaH* C_t_ <35
3. ipah C_t_ value < 27.9
4. Present at, or above, diarrhea associated quantity by TAC (n=697 [94%]); present below diarrhea-associated quantity by TAC (n=31 [4.2%]), absent by TAC (n=17 [2.3%])

| Table S4b. Other potential etiologies by diagnostic category | | | | | | | | | | | |
| --- | --- | --- | --- | --- | --- | --- | --- | --- | --- | --- | --- |
|  | | **Shigella**  **Culture** | **Absent** | | | | | | **Present** | | |
| **Characteristic** | | **Shigella**  **qPCR** | **Absent^i^**  **(n=3,397)** | | **qPCR-unattributable^ii^**  **(n=676)** | | **qPCR-attributable^iii^**  **(n=852)** | | **Any qPCR value^iv^**  **(n=745)** | | |
|  |  |  | aPR* | (95%CI) | aPR* | (95%CI) | aPR* | (95%CI) | | aPR* | (95%CI) |
| Viral | | |  |  |  |  |  |  | |  |  |
|  | Adenovirus | | 1.4 | (0.8-2.4) | 1.4 | (0.8-2.6) | 1.0 | (0.5-1.9) | | Ref | -- |
|  | Astrovirus | | 1.9 | (0.8-4.2) | 1.8 | (0.7-4.6) | 1.3 | (0.5-3.4) | | Ref | -- |
|  | Norovirus | | 1.3 | (0.6-3.0) | 0.6 | (0.2-2.0) | 0.9 | (0.3-2.6) | | Ref | -- |
|  | Rotavirus | | 3.4 | (2.5-4.6) | 2.7 | (1.9-3.8) | 1.0 | (0.7-1.5) | | Ref | -- |
|  | Sapovirus | | 1.1 | (0.5-2.3) | 1.3 | (0.5-3.0) | 0.9 | (0.4-2.1) | | Ref | -- |
| Parasitic | | |  |  |  |  |  |  | |  |  |
|  | *Cryptosporidium* | | 1.9 | (1.0-3.4) | 1.5 | (0.7-3.1) | 1.9 | (1.0-3.8) | | Ref | -- |
|  | *Cyclospora* | | NE | -- | NE | -- | NE | -- | | Ref | -- |
|  | *Entamoeba histolytica* | | NE | -- | NE | -- | NE | -- | | Ref | -- |
|  | *Isospora* | | NE | -- | NE | -- | NE | -- | | Ref | -- |
| Other bacteria | | |  |  |  |  |  |  | |  |  |
|  | *Aeromonas* | | 1.7 | (0.7-4.2) | 1.8 | (0.6-5.3) | 5.1 | (2.1-11.9) | | Ref | -- |
|  | *Campylobacter* | | NE | -- | NE | -- | NE | -- | | Ref | -- |
|  | EAEC | | NE | -- | NE | -- | NE | -- | | Ref | -- |
|  | *H. pylori* | | 1.1 | (0.6-2.0) | 1.2 | (0.6-2.4) | 1.5 | (0.8-3.0) | | Ref | -- |
|  | Lt-ETEC | | NE | -- | NE | -- | NE | -- | | Ref | -- |
|  | *Salmonella* | | 16.0 | (2.1-121.1) | 12.2 | (1.5-98.5) | 11.8 | (1.5-93.1) | | Ref | -- |
|  | STEC | | NE | -- | NE | -- | NE | -- | | Ref | -- |
|  | St-ETEC | | 2.2 | (1.4-3.7) | 3.1 | (1.8-5.2) | 2.3 | (1.4-4.0) | | Ref | -- |
|  | Typical EPEC | | 0.8 | (0.2-3.4) | 0.8 | (0.1-4.4) | 2.1 | (0.5-9.5) | | Ref | -- |
|  | *V. cholerae* | | 8.1 | (2.9-22.7) | 10.2 | (3.5-29.5) | 5.0 | (1.7-14.8) | | Ref | -- |

1. ipah C_t_ value ≥ 35
2. 27.9 ≤ *ipaH* C_t_ <35
3. ipah C_t_ value < 27.9
4. Present at, or above, diarrhea associated quantity by TAC (n=697 [94%]); present below diarrhea-associated quantity by TAC (n=31 [4.2%]), absent by TAC (n=17 [2.3%])

NE: Not estimable (ie too few data points in a given cell)

| Table S5. Clinical characteristics of PCR-attributable shigellosis (ipah CT value < 27.9) by age group (n=1549^i^) | | | | | | | | | |
| --- | --- | --- | --- | --- | --- | --- | --- | --- | --- |
| **Characteristic** | | **0-5m**  **(n=37)** | | **6-11m**  **(n=188)** | | **12-23m**  **(n=688)** | | **24-59m**  **(n=636)** | |
|  |  | n | (%) | n | (%) | n | (%) | n | (%) |
| **Sociodemographic** | |  |  |  |  |  |  |  |  |
|  | Female | 16 | (43.2) | 73 | (38.8) | 317 | (46.1) | 283 | (44.5) |
|  | Site |  |  |  |  |  |  |  |  |
|  | Bangladesh | 3 | (8.1) | 43 | (22.9) | 193 | (28.1) | 245 | (38.5) |
|  | India | 4 | (10.8) | 18 | (9.6) | 64 | (9.3) | 106 | (16.7) |
|  | Kenya | 8 | (21.6) | 25 | (13.3) | 56 | (8.1) | 59 | (9.3) |
|  | Mali | 1 | (2.7) | 19 | (10.1) | 95 | (13.8) | 65 | (10.2) |
|  | Mozambique | 0 | (0) | 11 | (5.9) | 54 | (7.9) | 42 | (6.6) |
|  | Pakistan | 19 | (51.4) | 37 | (19.7) | 111 | (16.3) | 67 | (10.5) |
|  | The Gambia | 2 | (5.4) | 35 | (18.6) | 115 | (16.7) | 52 | (8.2) |
| **Clinical characteristics** | |  |  |  |  |  |  |  |  |
|  | Observed or caregiver reported blood in stool | 12 | (32.4) | 92 | (48.9) | 361 | (52.5) | 401 | (63.1) |
|  | Caregiver reported mucoid stool | 12 | (32.4) | 70 | (37.2) | 261 | (37.9) | 256 | (40.3) |
|  | Duration of diarrhea (including day of presentation) |  |  |  |  |  |  |  |  |
|  | 1-3 | 20 | (54.1) | 134 | (71.3) | 485 | (70.5) | 463 | (72.8) |
|  | 4-5 | 14 | (37.8) | 34 | (18.1) | 152 | (22.1) | 135 | (21.2) |
|  | 6+ | 3 | (8.1) | 20 | (10.6) | 51 | (7.4) | 38 | (6.0) |
|  | Max # of loose stools child passed in 24 hour period |  |  |  |  |  |  |  |  |
|  | ≤6 | 17 | (46.0) | 98 | (52.1) | 383 | (55.7) | 348 | (54.7) |
|  | 7-10 | 12 | (32.4) | 64 | (34.0) | 205 | (29.8) | 185 | (29.1) |
|  | >10 | 8 | (21.6) | 26 | (13.8) | 100 | (14.5) | 103 | (16.2) |
|  | Axillary temperature at presentation |  |  |  |  |  |  |  |  |
|  | <38°C | 31 | (83.8) | 153 | (81.4) | 543 | (78.9) | 456 | (71.7) |
|  | 38-38.9°C | 5 | (13.5) | 26 | (13.8) | 82 | (11.9) | 106 | (16.7) |
|  | ≥39°C | 1 | (2.7) | 9 | (4.8) | 63 | (9.2) | 74 | (11.6) |
|  | Caregiver reported vomiting ≥ 3 times per day | 15 | (40.5) | 62 | (33.0) | 169 | (24.6) | 127 | (20.0) |
|  | WHO-defined dehydration categories |  |  |  |  |  |  |  |  |
|  | None | 10 | (27.0) | 51 | (27.1) | 212 | (30.8) | 259 | (40.7) |
|  | Some | 4 | (10.8) | 44 | (23.4) | 177 | (25.7) | 151 | (23.7) |
|  | Severe | 23 | (62.2) | 93 | (49.5) | 299 | (43.5) | 226 | (35.5) |
|  | Modified Vesikari Score^ii^ |  |  |  |  |  |  |  |  |
|  | Mild | 4 | (10.8) | 24 | (12.8) | 82 | (11.9) | 98 | (15.4) |
|  | Moderate | 16 | (43.2) | 80 | (42.6) | 344 | (50.0) | 315 | (49.5) |
|  | Severe | 17 | (46.0) | 84 | (44.7) | 262 | (38.1) | 223 | (35.1) |
|  | Stunted (LAZ<-2) | 14 | (37.8) | 46 | (24.7) | 218 | (31.9) | 233 | (36.9) |
|  | Wasted (MUAC < 12.5cm)^iii^ | NA | NA | 53 | (28.2) | 101 | (14.7) | 30 | (4.7) |
| ***Shigella*** **lab results** | |  |  |  |  |  |  |  |  |
|  | Culture positive | 14 | (37.8) | 62 | (33.0) | 283 | (41.1) | 338 | (53.1) |
| iAmong children with qPCR-attributable *Shigella* (which comprised of 852 qPCR-attributable/culture negative and 697 qPCR-attributable/ culture positive. Did not exclude based on lack of vital status information).  i^i^As derived in Kotloff et al., Vaccine, 2017  ^iii^ Among children older than 6 months | | | | | | | | | |

| Table S6. Clinical characteristics of culture-confirmed shigellosis by age group (n=745) | | | | | | | | | |
| --- | --- | --- | --- | --- | --- | --- | --- | --- | --- |
| **Characteristic** | | **0-5m**  **(n=18)** | | **6-11m**  **(n=73)** | | **12-23m**  **(n=301)** | | **24-59m**  **(n=353)** | |
|  |  | n | (%) | n | (%) | n | (%) | n | (%) |
| **Sociodemographic** | |  |  |  |  |  |  |  |  |
|  | Female | 9 | (50.0) | 22 | (30.1) | 150 | (49.8) | 158 | (44.8) |
|  | Site |  |  |  |  |  |  |  |  |
|  | Bangladesh | 3 | (16.7) | 33 | (45.2) | 160 | (53.2) | 214 | (60.6) |
|  | India | 3 | (16.7) | 3 | (4.1) | 18 | (6.0) | 34 | (9.6) |
|  | Kenya | 6 | (33.3) | 10 | (13.7) | 20 | (6.6) | 36 | (10.2) |
|  | Mali | 0 | (0) | 0 | (0) | 11 | (3.7) | 7 | (1.2) |
|  | Mozambique | 1 | (5.6) | 2 | (2.7) | 8 | (2.7) | 13 | (3.7) |
|  | Pakistan | 5 | (27.8) | 15 | (20.6) | 40 | (13.3) | 26 | (7.4) |
|  | The Gambia | 0 | (0) | 10 | (13.7) | 44 | (14.6) | 23 | (6.5) |
| **Clinical characteristics** | |  |  |  |  |  |  |  |  |
|  | Observed or caregiver reported blood in stool | 6 | (33.3) | 51 | (69.9) | 212 | (70.4) | 265 | (75.1) |
|  | Caregiver reported mucoid stool | 7 | (38.9) | 33 | (45.2) | 145 | (48.2) | 179 | (50.7) |
|  | Duration of diarrhea (including day of presentation) |  |  |  |  |  |  |  |  |
|  | 1-3 | 13 | (72.2) | 47 | (64.4) | 214 | (71.1) | 267 | (75.6) |
|  | 4-5 | 4 | (22.2) | 15 | (20.6) | 64 | (21.3) | 66 | (18.7) |
|  | 6+ | 1 | (5.6) | 11 | (15.1) | 23 | (7.6) | 20 | (5.7) |
|  | Max # of loose stools child passed in 24 hour period |  |  |  |  |  |  |  |  |
|  | ≤6 | 10 | (55.6) | 29 | (39.7) | 124 | (41.2) | 158 | (44.8) |
|  | 7-10 | 4 | (22.2) | 29 | (39.7) | 99 | (32.9) | 115 | (32.6) |
|  | >10 | 4 | (22.2) | 15 | (20.6) | 78 | (25.9) | 80 | (22.7) |
|  | Axillary temperature at presentation |  |  |  |  |  |  |  |  |
|  | <38°C | 14 | (77.8) | 54 | (74.0) | 222 | (73.8) | 217 | (61.5) |
|  | 38-38.9°C | 2 | (11.1) | 14 | (19.2) | 39 | (13.0) | 73 | (20.7) |
|  | ≥39°C | 2 | (11.1) | 5 | (6.9) | 40 | (13.3) | 63 | (17.9) |
|  | Caregiver reported vomiting ≥ 3 times per day | 7 | (38.9) | 18 | (24.7) | 65 | (21.6) | 72 | (20.4) |
|  | WHO-defined dehydration categories |  |  |  |  |  |  |  |  |
|  | None | 7 | (38.9) | 28 | (38.4) | 136 | (45.2) | 187 | (53.0) |
|  | Some | 4 | (22.2) | 20 | (27.4) | 67 | (22.3) | 75 | (21.3) |
|  | Severe | 7 | (38.9) | 25 | (34.3) | 98 | (32.6) | 91 | (25.8) |
|  | Modified Vesikari Score^i^ |  |  |  |  |  |  |  |  |
|  | Mild | 4 | (22.2) | 9 | (12.3) | 44 | (14.6) | 56 | (15.9) |
|  | Moderate | 8 | (44.4) | 33 | (45.2) | 130 | (43.2) | 157 | (44.5) |
|  | Severe | 6 | (33.3) | 31 | (42.5) | 127 | (42.2) | 140 | (39.7) |
|  | Stunted (LAZ<-2) | 3 | (16.7) | 17 | (23.9) | 84 | (27.9) | 110 | (31.3) |
|  | Wasted (MUAC < 12.5cm)^ii^ | NA | NA | 13 | (17.8) | 40 | (13.3) | 13 | (3.4) |
| ***Shigella*** **lab results** | |  |  |  |  |  |  |  |  |
|  | qPCR attributable | 14 | (77.8) | 62 | (84.9) | 283 | (94.0) | 338 | (95.8) |
| iAs derived in Kotloff et al., Vaccine, 2017  i^i^Among children older than 6 months | | | | | | | | | |

| Table S7. Characteristics of GEMS *Shigella* MSD cases (N=1,044) who died vs. those who survived in the 14 days post enrollment removing children with a possible etiology (AF>.5) other than *Shigella* in the two likely *Shigella* categories (*Shigella* culture positive or *Shigella* culture positive/ molecular attributable). | | | | | | | |
| --- | --- | --- | --- | --- | --- | --- | --- |
| **Characteristic** | | **Died**  **(n=14)** | | **Survived**  **(n=1,030)** | | **Hazard Ratio^ii^ (95% CI)** | **aHazard Ratio (95% CI)^iii^** |
|  |  | n | (%)^i^ | n | (%)^i^ |  |  |
| **Sociodemographic** | |  |  |  |  |  |  |
|  | Age |  |  |  |  |  |  |
|  | 0m to 5m | 1 | (7.1) | 22 | (2.1) | 7.0 (1.4-34.6) | 4.5 (0.9-23.2) |
|  | 6m to 11m | 3 | (21.4) | 110 | (10.7) | 4.8 (1.6-14.3) | 3.6 (1.2-10.8) |
|  | 12m to 23m | 6 | (42.9) | 451 | (43.8) | 1.8 (0.7-4.9) | 1.4 (0.5-3.8) |
|  | 24m to 59m | 4 | (28.6) | 447 | (43.4) | Ref | Ref |
|  | Sex |  |  |  |  |  |  |
|  | Female | 4 | (28.6) | 462 | (44.9) | 0.8 (0.4-1.7) | 0.8 (0.3-1.7) |
|  | Male | 10 | (71.4) | 568 | (55.1) | Ref | Ref |
| **Clinical characteristics** | |  |  |  |  |  |  |
|  | Dysentery |  |  |  |  |  |  |
|  | Present | 6 | (42.9%) | 636 | (61.8) | 0.3 (0.1-0.7) | 0.6 (0.2-1.3) |
|  | Absent | 8 | (57.1%) | 394 | (38.3) | Ref | Ref |
|  | Caregiver reported mucoid stool |  |  |  |  |  |  |
|  | Present | 5 | (35.7) | 438 | (42.5) | 1.0 (0.5-2.2) | 1.8 (0.8-4) |
|  | Absent | 9 | (64.3) | 592 | (57.5) | Ref | Ref |
|  | Duration of diarrhea (including day of presentation) |  |  |  |  |  |  |
|  | ≥3 | 11 | (78.6) | 520 | (50.5) | 3.3 (1.3-8.2) | 3.0 (1.2-7.6) |
|  | <3 | 3 | (21.4) | 510 | (49.5) | Ref | Ref |
|  | Max # of loose stools child passed in 24 hour period |  |  |  |  |  |  |
|  | ≥7 | 4 | (28.6) | 470 | (45.6) | 0.4 (0.2-1) | 0.8 (0.3-2.1) |
|  | <7 | 10 | (71.4) | 560 | (54.4) | Ref | Ref |
|  | Temperature |  |  |  |  |  |  |
|  | ≥38°C | 8 | (57.1) | 272 | (26.4) | 2.4 (1.1-5.1) | 2.5 (1.1-5.5) |
|  | <38°C | 6 | (42.9) | 758 | (73.6) | Ref | Ref |
|  | Caregiver reported vomiting |  |  |  |  |  |  |
|  | > 3 times per day | 8 | (57.1) | 215 | (20.9) | 8.6 (3.7-19.8) | 6.4 (2.8-15) |
|  | ≤3 times per day (or none) | 6 | (42.9) | 815 | (79.1) | Ref | Ref |
|  | WHO-defined dehydration categories |  |  |  |  |  |  |
|  | Severe | 13 | (92.9) | 391 | (38.0) | 8.1 (2.4-26.9) | 2.6 (0.6-10.3) |
|  | Some | 0 | (0) | 241 | (23.4) | Not estimable | Not estimable |
|  | None | 1 | (7.1) | 398 | (38.6) | Ref | Ref |
|  | Chronic Malnutrition |  |  |  |  |  |  |
|  | Stunted (LAZ<-2) | 7 | (50) | 330 | (32) | 6.0 (2.4-15.3) | 6.3 (2.5-16.2) |
|  | Non-stunted | 5 | (35.7) | 696 | (67.6) | Ref | Ref |
|  | Acute malnutrition ^iv^ |  |  |  |  |  |  |
|  | MUAC < 12.5cm | 7 | (50) | 114 | (11.1) | 13.0 (5.9-28.6) | 10.4 (4.4-24.6) |
|  | MUAC≥ 12.5cm | 7 | (50) | 916 | (88.9) | Ref | Ref |
|  | Admission status at enrollment visit |  |  |  |  |  |  |
|  | Hospitalized | 11 | (78.6) | 216 | (21) | 5.9 (2.7-13.0) | 9.9 (3.9-24.9) |
|  | Seen as outpatient | 3 | (21.4) | 814 | (79) | Ref | Ref |
|  | Modified Vesikari Score ^v^ |  |  |  |  |  |  |
|  | Severe | 12 | (85.7) | 360 | (35.0) | 5.8 (2.2-15.4) | 4.9 (1.8-13.1) |
|  | Moderate | 2 | (14.3) | 520 | (50.5) | Ref | Ref |
|  | Mild | 0 | (0) | 150 | (14.6) | Not estimable | Not estimable |
| **Laboratory** | |  |  |  |  |  |  |
|  | Shigella culture results |  |  |  |  |  |  |
|  | Culture positive | 7 | (50) | 545 | (52.9) | 0.6 (0.3-1.4) | 1.0 (0.4-2.2) |
|  | Culture negative | 7 | (50) | 485 | (47.1) | Ref | Ref |

1. Column percentages
2. From Cox proportional hazards regression including only the variable of interest in the model
3. From Cox proportional hazards regression including the variable of interest, site as an indicator variable, and age as a continuous variable except age model adjusted only for site
4. Among those≥6 months of age in whom MUAC
5. As derived in Kotloff et al., Vaccine, 2017

| Table S8. Characteristics of GEMS *Shigella* MSD cases (N=979) who died vs. those who survived in the 14 days post enrollment removing Bangladesh site because of unique *Shigella* epidemiology | | | | | | | |
| --- | --- | --- | --- | --- | --- | --- | --- |
| **Characteristic** | | **Died**  **(n=21)** | | **Survived**  **(n=958)** | | **Hazard Ratio^ii^ (95% CI)** | **aHazard Ratio (95% CI)^iii^** |
|  |  | n | (%)^i^ | n | (%)^i^ |  |  |
| **Sociodemographic** | |  |  |  |  |  |  |
|  | Age |  |  |  |  |  |  |
|  | 0m to 5m | 3 | (14.3) | 29 | (3) | 8.6 (1.9-38.6) | 7.3 (1.6-33.7) |
|  | 6m to 11m | 4 | (19) | 132 | (13.8) | 2.6 (0.7-10.5) | 1.8 (0.5-7.4) |
|  | 12m to 23m | 10 | (47.6) | 446 | (46.6) | 2 (0.6-6.2) | 1.5 (0.5-5) |
|  | 24m to 59m | 4 | (19) | 351 | (36.6) | Ref | Ref |
|  | Sex |  |  |  |  |  |  |
|  | Female | 7 | (33.3) | 436 | (45.5) | 0.6 (0.2-1.5) | 0.6 (0.2-1.5) |
|  | Male | 14 | (66.7) | 522 | (54.5) | Ref | Ref |
| **Clinical characteristics** | |  |  |  |  |  |  |
|  | Dysentery |  |  |  |  |  |  |
|  | Present | 6 | (28.6%) | 385 | (40.2%) | 0.5 (0.3-1.1) | 0.5 (0.3-1.1) |
|  | Absent | 15 | (71.4%) | 573 | (59.8%) | Ref | Ref |
|  | Caregiver reported mucoid stool |  |  |  |  |  |  |
|  | Present | 7 | (33.3) | 240 | (25.1) | 1.5 (0.6-3.7) | 1.1 (0.4-2.9) |
|  | Absent | 14 | (66.7) | 718 | (74.9) | Ref | Ref |
|  | Duration of diarrhea (including day of presentation) |  |  |  |  |  |  |
|  | ≥3 | 19 | (90.5) | 503 | (52.5) | 8.4 (2.0-36.2) | 6.6 (1.5-29.4) |
|  | <3 | 2 | (9.5) | 455 | (47.5) | Ref | Ref |
|  | Max # of loose stools child passed in 24 hour period |  |  |  |  |  |  |
|  | ≥7 |  |  |  |  | 0.7 (0.3-1.8) | 1 (0.4-2.8) |
|  | <7 | 15 | (71.4) | 606 | (63.3) | Ref | Ref |
|  | Temperature |  |  |  |  |  |  |
|  | ≥38°C | 8 | (38.1) | 174 | (18.2) | 2.7 (1.1-6.6) | 1.9 (0.8-4.8) |
|  | <38°C | 13 | (61.9) | 784 | (81.8) | Ref | Ref |
|  | Caregiver reported vomiting |  |  |  |  |  |  |
|  | > 3 times per day | 12 | (57.1) | 253 | (26.4) | 3.7 (1.5-8.7) | 2.7 (1.1-6.5) |
|  | ≤3 times per day (or none) | 9 | (42.9) | 705 | (73.6) | Ref | Ref |
|  | WHO-defined dehydration categories |  |  |  |  |  |  |
|  | Severe | 19 | (90.5) | 538 | (56.2) | 5.3 (0.7-39.7) | 3.8 (0.5-29.3) |
|  | Some | 1 | (4.8) | 267 | (27.9) | 0.6 (0-9.2) | 0.7 (0-12.4) |
|  | None | 1 | (4.8) | 153 | (16) | Ref | Ref |
|  | Chronic Malnutrition |  |  |  |  |  |  |
|  | Stunted (LAZ<-2) | 11 | (61.1) | 326 | (34.2) | 3.0 (1.2-7.7) | 3.5 (1.3-9.1) |
|  | Non-stunted | 7 | (38.9) | 627 | (65.8) | Ref | Ref |
|  | Acute malnutrition ^iv^ |  |  |  |  |  |  |
|  | MUAC < 12.5cm | 8 | (38.1) | 143 | (14.9) | 4.3 (1.7-10.8) | 3.4 (1.2-9.0) |
|  | MUAC≥ 12.5cm | 10 | (47.6) | 786 | (82) | Ref | Ref |
|  | Admission status at enrollment visit |  |  |  |  |  |  |
|  | Hospitalized | 15 | (71.4) | 152 | (15.9) | 12.7 (4.9-32.6) | 14.0 (4.9-40.1) |
|  | Seen as outpatient | 6 | (28.6) | 806 | (84.1) | Ref | Ref |
|  | Modified Vesikari Score ^v^ |  |  |  |  |  |  |
|  | Severe | 17 | (81) | 368 | (38.4) | 5.7 (1.9-16.8) | 4.1 (1.4-12.1) |
|  | Moderate | 4 | (19) | 500 | (52.2) | Ref | Ref |
|  | Mild | 0 | (0) | 90 | (9.4) | Not estimable | Not estimable |
| **Laboratory** | |  |  |  |  |  |  |
|  | Shigella culture results |  |  |  |  |  |  |
|  | Culture positive | 9 | (42.9) | 292 | (30.5) | 1.7 (0.7-4) | 1.3 (0.5-3) |
|  | Culture negative | 12 | (57.1) | 666 | (69.5) | Ref | Ref |
|  | Other potential etiology^vi^ |  |  |  |  |  |  |
|  | Yes | 8 | (38.1) | 316 | (33) | 1.2 (0.5-3.0) | 1.4 (0.6-3.5) |
|  | No | 13 | (61.9) | 642 | (67) | Ref | Ref |

1. Column percentages
2. From Cox proportional hazards regression including only the variable of interest in the model
3. From Cox proportional hazards regression including the variable of interest, site as an indicator variable, and age as a continuous variable except age model adjusted only for site
4. Among those≥6 months of age in whom MUAC is validated
5. As derived in Kotloff et al., Vaccine, 2017
6. Based on site and age-adjusted attributable fraction ≥.5 for any of the following pathogens: astrovirus, norovirus, rotavirus, sapovirus, adenovirus, *Cryptosporidium*, *E. histolytica*, *Cyclospora*, *Isospora, H. pylori*, *Salmonella*, *V. cholerae*, EAEC, St-ETEC, Lt-ETEC, tEPEC, STEC

| Table S9. Characteristics of GEMS *Shigella* MSD cases (N=1,467) who died vs. those who survived in the 14 days post enrollment removing those who were not part of the random subset selected for molecular analysis (aka enriched fatal cases) | | | | | | | |
| --- | --- | --- | --- | --- | --- | --- | --- |
| **Characteristic** | | **Died**  **(n=16)** | | **Survived**  **(n=1,451)** | | **Hazard Ratio^ii^ (95% CI)** | **aHazard Ratio (95% CI)^iii^** |
|  |  | n | (%)^i^ | n | (%)^i^ |  |  |
| **Sociodemographic** | |  |  |  |  |  |  |
|  | Age |  |  |  |  |  |  |
|  | 0m to 5m | 1 | (6.3) | 31 | (2.1) | 3.8 (0.4-32.8) | 2.0 (0.2-18.2) |
|  | 6m to 11m | 3 | (18.8) | 177 | (12.2) | 2.0 (0.5-8.5) | 1.3 (0.3-5.4) |
|  | 12m to 23m | 7 | (43.8) | 641 | (44.2) | 1.3 (0.4-4.1) | 1.0 (0.3-3.2) |
|  | 24m to 59m | 5 | (31.3) | 602 | (41.5) | Ref | Ref |
|  | Sex |  |  |  |  |  |  |
|  | Female | 6 | (37.5) | 648 | (44.7) | 0.7 (0.3-2.1) | 0.7 (0.3-1.9) |
|  | Male | 10 | (62.5) | 803 | (55.3) | Ref | Ref |
| **Clinical characteristics** | |  |  |  |  |  |  |
|  | Dysentery |  |  |  |  |  |  |
|  | Present | 5 | (31.3%) | 828 | (57.1%) | 0.3 (0.1-1.0) | 0.6 (0.2-1.7) |
|  | Absent | 11 | (68.8%) | 623 | (42.9%) | Ref | Ref |
|  | Caregiver reported mucoid stool |  |  |  |  |  |  |
|  | Present | 5 | (68.8) | 572 | (39.4) | 0.7 (0.2-2) | 0.9 (0.3-2.6) |
|  | Absent | 11 | (0) | 879 | (60.6) | Ref | Ref |
|  | Duration of diarrhea (including day of presentation) |  |  |  |  |  |  |
|  | ≥3 | 13 | (81.3) | 751 | (51.8) | 4 (1.1-14.1) | 3.4 (0.9-12.3) |
|  | <3 | 3 | (18.8) | 700 | (48.2) | Ref | Ref |
|  | Max # of loose stools child passed in 24 hour period |  |  |  |  |  |  |
|  | ≥7 | 6 | (37.5) | 675 | (46.5) | 0.7 (0.3-1.9) | 1.4 (0.5-4.0) |
|  | <7 | 10 | (62.5) | 776 | (53.5) | Ref | Ref |
|  | Temperature |  |  |  |  |  |  |
|  | ≥38°C | 6 | (37.5) | 347 | (23.9) | 1.9 (0.7-5.2) | 1.8 (0.6-5.0) |
|  | <38°C | 10 | (62.5) | 1104 | (76.1) | Ref | Ref |
|  | Caregiver reported vomiting |  |  |  |  |  |  |
|  | > 3 times per day | 9 | (56.3) | 339 | (23.4) | 4.2 (1.6-11.2) | 3.0 (1.1-8.1) |
|  | ≤3 times per day (or none) | 7 | (43.8) | 1112 | (76.6) | Ref | Ref |
|  | WHO-defined dehydration categories |  |  |  |  |  |  |
|  | Severe | 14 | (87.5) | 570 | (39.3) | 12.7 (1.7-96.8) | 6 (0.6-65.1) |
|  | Some | 1 | (6.3) | 356 | (24.5) | 1.5 (0.1-23.6) | 1.3 (0.1-24) |
|  | None |  |  |  |  | Ref | Ref |
|  | Chronic Malnutrition |  |  |  |  |  |  |
|  | Stunted (LAZ<-2) | 8 | (50) | 460 | (31.7) | 3.4 (1.1-10.4) | 3.5 (1.1-10.9) |
|  | Non-stunted | 5 | (31.3) | 987 | (68) | Ref | Ref |
|  | Acute malnutrition ^iv^ |  |  |  |  |  |  |
|  | MUAC < 12.5cm | 7 | (43.8) | 177 | (12.2) | 5.5 (2-14.7) | 3.8 (1.3-11.2) |
|  | MUAC≥ 12.5cm | 9 | (56.3) | 1274 | (87.8) | Ref | Ref |
|  | Admission status at enrollment visit |  |  |  |  |  |  |
|  | Hospitalized | 13 | (81.3) | 315 | (21.7) | 15.3 (4.4-53.8) | 30.4 (7.5-123.4) |
|  | Seen as outpatient | 3 | (18.8) | 1136 | (78.3) | Ref | Ref |
|  | Modified Vesikari Score ^v^ |  |  |  |  |  |  |
|  | Severe | 13 | (81.3) | 535 | (36.9) | 5.7 (1.6-20.1) | 4.3 (1.2-15.3) |
|  | Moderate | 3 | (18.8) | 716 | (49.4) | Ref | Ref |
|  | Mild | 0 | (0) | 200 | (13.8) | Not estimable | Not estimable |
| **Laboratory** | |  |  |  |  |  |  |
|  | Shigella culture results |  |  |  |  |  |  |
|  | Culture positive | 6 | (37.5) | 696 | (48) | 0.7 (0.2-1.8) | 0.8 (0.3-2.4) |
|  | Culture negative | 10 | (62.5) | 755 | (52) | Ref | Ref |
|  | Other potential etiology ^vi^ |  |  |  |  |  |  |
|  | Yes | 7 | (43.8) | 428 | (29.5) | 1.8 (0.7-5) | 1.9 (0.7-5.1) |
|  | No | 9 | (56.3) | 1023 | (70.5) | Ref | Ref |

1. Column percentages
2. From Cox proportional hazards regression including only the variable of interest in the model
3. From Cox proportional hazards regression including the variable of interest, site as an indicator variable, and age as a continuous variable except age model adjusted only for site
4. Among those≥6 months of age in whom MUAC is validated
5. As derived in Kotloff et al., Vaccine, 2017
6. Based on site and age-adjusted attributable fraction ≥.5 for any of the following pathogens: astrovirus, norovirus, rotavirus, sapovirus, adenovirus, *Cryptosporidium*, *E. histolytica*, *Cyclospora*, *Isospora, H. pylori*, *isospora*, *Salmonella*, *V. cholerae*, EAEC, St-ETEC, Lt-ETEC, tEPEC, STEC

| **Table S10. *Shigella* risk score point value assignment based on cox proportional hazards model betas using death in last 14 days as the outcome and constraining the maximum number of points to be 16 to match the modified Vesikari score** | | | | | | | | | |
| --- | --- | --- | --- | --- | --- | --- | --- | --- | --- |
| **Predictor** | | **Reference Value (W*_ij_*)** | **Referent Group (W*_iREF_*)** | **β*_i_*** | **(W*_ij_* – W*_iREF_*)** | **β_i (_W*_ij_* _–_ W*_iREF_*_)_** | **B** | **Points [βi(W*_ij_* -W*_iREF_*)/B]** |  |
|  |  |  |  |  |  |  | 0.50 |  |  |
| **Duration of diarrhea**  **(including day of presentation)** | |  |  | 0.629 |  |  |  |  |  |
|  | ≥6 | 2 |  |  | 2 | 1.259 |  | 3 |  |
|  | 4-5 | 1 |  |  | 1 | 0.629 |  | 2 |  |
|  | 1-3 | 0 | Ref |  | 0 | 0 |  | 0 |  |
| **WHO-defined dehydration categories** | |  |  | 1.563 |  |  |  |  |  |
|  | Severe | 2 |  |  | 2 | 3.125 |  | 8 |  |
|  | Some | 1 |  |  | 1 | 1.563 |  | 4 |  |
|  | None | 0 | Ref |  | 0 | 0 |  | 0 |  |
| **Clinician decision to hospitalize** | |  |  | 2.151 |  |  |  |  |  |
|  | Yes | 1 |  |  | 1 | 2.151 |  | 5 |  |
|  | No | 0 | Ref |  | 0 | 0 |  | 0 |  |

Figure S1. ROC curves of model-derived *Shigella* severity score & modified Vesikari score predicting death in first 14-days and within 60-days (range 50-90 days) among 14-day survivors in the subset of 1, 261 children aged 12 months and older. *Shigella* model-derived severity score: AUC_0-14_ of 0.82 (95% CI: 0.71-0.89); AUC_15-90_ 0.79 (95%CI: 0.60-0.90). Modified Vesikari score: AUC_0-14:_ 0.75 (95%CI: 0.63-0.87); AUC_15-90_: 0.68 (95% CI: 0.52-0.82).AUC comparisons: 0-14 days (p-value_AUC derived vs. AUC MVS_ = 0.048) and 15-90 days (p-value_AUC derived vs. AUC MVS_ = 0.082)

0-14 days

15-90 days

**Modified Vesikari score**

**Model-derived *Shigella***

**severity score**

0-14 days

15-90 days

Figure S2. ROC curves of model-derived *Shigella* severity score & modified Vesikari score predicting death in first 14-days and within 60-days (range 50-90 days) among 14-day survivors in the subset of 707 *Shigella* culture positive cases). *Shigella* model-derived severity score: AUC_0-14_ of 0.83 (95% CI: 0.69-0.94); AUC_15-90_ 0.89 (95%CI: 0.74-0.97). Modified Vesikari score: AUC_0-14:_ 0.77 (95%CI: 0.59-0.93); AUC_15-90_: 0.74 (95% CI: 0.43-0.93). AUC comparisons: 0-14 days (p-value_AUC derived vs. AUC MVS_ = 0.174) and 15-90 days (p-value_AUC derived vs. AUC MVS_ = 0.174)

0-14 days

15-90 days

**Modified Vesikari score**

**Model-derived *Shigella***

**severity score**

0-14 days

15-90 days
